# Evolving Dynamics of Colorectal Cancer in High Socio-Demographic Regions

**DOI:** 10.1177/10732748251321672

**Published:** 2025-02-17

**Authors:** Laalithya Konduru, Simranjeet Singh Dahia, Claudia Szabo, Savio G. Barreto

**Affiliations:** 1 College of Medicine and Public Health, 198094 Flinders University, Bedford Park, SA, Australia; 2 Healthynfinity Pvt Ltd, Chennai, India; 3 School of Computer and Mathematical Sciences, 549440 University of Adelaide, Adelaide, SA, Australia; 4 Division of Surgery and Perioperative Medicine, 14351 Flinders Medical Centre, Bedford Park, SA, Australia

**Keywords:** colorectal cancer, disease forecasting, epidemiological shift, healthcare planning, machine learning

## Abstract

**Background:**

Colorectal cancer (CRC) poses a significant global health challenge, with evolving demographic trends emphasizing the need for accurate forecasting models. Existing forecasting models lack comprehensive coverage. By integrating machine learning algorithms, this study aims to provide more accurate and precise predictions, filling critical gaps in understanding CRC incidence, death, and disability-adjusted life year (DALY) rate trends, especially in high socio-demographic index (SDI) regions. Specific emphasis is placed on exploring age-, sex-, and country-specific variations in CRC trends.

**Materials and Methods:**

An ensemble forecasting algorithm integrating Simple Linear Regression, Exponential Smoothing, and Autoregressive Integrated Moving Average, capable of handling a short time series was developed and validated, utilizing a dataset encompassing age-, sex-, and country-specific CRC incidence, mortality, and DALY rates.

**Results:**

Our forecasting models reveal rising trends in CRC burden in the 15-49 years age group (young-onset) and decreasing trends in CRC burden in the 50-74 years age group (late-onset) in high SDI regions with sex-specific variations in incidence, mortality, and DALY rates. Some inflection points for demographic shifts in CRC disease burden, particularly death rates, were identified as early as within the next 5 years. We predict a shift in CRC burden towards females, particularly in older adults.

**Conclusion:**

A novel multifactor model was developed for comparing the incidence, mortality, and DALY rates of young- and late-onset CRC in high SDI regions. The rising incidence of young-onset CRC in high SDI regions underscores the need for proactive health strategies. By refining predictive models, adjusting screening guidelines to target younger, high-risk populations, and investing in public awareness and research, we can facilitate early detection and improve outcomes. This study addresses a significant gap in CRC forecasting and provides a robust framework for anticipating demographic shifts in CRC burden, making it an indispensable tool for healthcare planning.

## Plain Language Summary

Colorectal cancer (CRC) is a growing global health concern, and understanding how its burden changes over time is essential for healthcare planning. Forecasting models are used to predict future trends in CRC, but existing models are often limited in scope and accuracy. This study aims to improve those predictions by using machine learning algorithms to provide more precise forecasts. The model focuses on predicting CRC incidence, death, and disability-adjusted life year (DALY; a measure of overall disease burden, with 1 DALY representing the loss of the equivalent of 1 year of good health) rates, with a special emphasis on high socio-demographic index (SDI; a composite metric that identifies where countries or other geographic areas lie on the spectrum of development based on per capita income, average years of schooling, and total fertility rate) regions—countries with better healthcare systems and economic conditions. Our model looks at how CRC affects different groups of people based on their age, sex, and country. The results highlight an increase in CRC among younger individuals aged 15 to 49, while showing a decline among those aged 50 to 74 in high SDI regions. Interestingly, we predict that the burden of CRC will shift toward females, particularly in the older age group, over the next few years. These demographic shifts will be crucial for healthcare systems to consider. The study’s machine learning-based model performs better than traditional models in predicting CRC trends. It identifies specific points in time when significant changes in disease patterns are expected, helping healthcare providers prepare for future needs. Overall, this research offers a new and powerful tool to forecast CRC burden and guide public health strategies.

## Introduction

Cancer poses a significant public health threat and places a substantial economic burden on healthcare systems.^1^ Globally, among the top 15 causes of death, cancer is the leading cause of financial strain, with an estimated expenditure of 232.9 billion USD (95% uncertainty interval: 85.9-422.0 billion USD) from 2020 to 2030—a 6.9% increase in treatment costs.^2^ Consequently, developing cancer burden projection models is essential for policymakers to effectively allocate resources for cancer prevention and healthcare planning. The accuracy of such projections depends on the predictive models used and their ability to align closely with future observed cancer rates.

The reliability of forecasting models is critical for ensuring accurate predictions and effective decision-making. While model fitting parameters such as the *r*^2^ statistic are commonly reported, they are not a measure of a model’s predictive accuracy or its ability to handle unseen data. The *r*^2^ measures the proportion of variance explained by the model and how well the model performs on the training data, but does not indicate whether the model is overfitting—capturing noise in the data rather than the true underlying relationship.^3^ In contrast, performance metrics like Mean Absolute Error (MAE), Mean Squared Error (MSE), Root MSE (RMSE), and Normalized Root MSE (NRMSE) offer a more robust evaluation by quantifying the model’s actual prediction error and assessing its generalizability.^3,4^ These metrics help policymakers determine if the model performs well not only on training data but also on test or validation data, which can reveal overfitting.^3,4^ Unfortunately, most cancer forecasting studies, including those from the Global Burden of Disease (GBD)^5^ and Global Cancer Observatory (GLOBOCAN),^6^ do not report the performance metrics of their models.

Various models have been developed to project cancer incidence trends, including the joinpoint model,^7^ delay-adjusted average annual percentage change (AAPC),^8^ and autoregressive integrated moving average (ARIMA) models and their variants.^9,10^ Long-term projections are critical for strategic decision-making in cancer prevention and treatment. Furthermore, any impending epidemiological shifts are difficult to predict in the short term but are more likely to emerge over the medium to long term. However, most studies using easy-to-use statistical methods like the joinpoint model and AAPC focus on short-term projections (3-5 years).^11^ Some complex models, such as age-period-cohort models, provide medium-term forecasts (up to 15 years).^11^ The GBD Foresight^5^ and GLOBOCAN Cancer Tomorrow^6^ extend the forecasts to 22 and 28 years, respectively, but the absence of performance metrics limits the ability to fully assess their robustness.^11^ Machine learning (ML) models may be simpler to use and can provide long-term forecasts, however, the small size of cancer epidemiological datasets often limits the use of advanced ML models, which are prone to overfitting and high variance.^12,13^ Thus, there is a pressing need for simpler, reliable models, with known performance metrics, purpose-built for providing accurate long-term forecasts to support healthcare planning and resource allocation. This study aims to develop such a model—efficient yet robust—offering long-term cancer projections without requiring complex mathematical data engineering. Given the significant global burden and high mortality rate of colorectal cancer (CRC) among gastrointestinal cancers, we have chosen to model CRC, highlighting the need for improved predictive models.

CRC, with almost 2 000 000 cases diagnosed worldwide in 2020 and an estimated 1 000 000 deaths per year,^14,15^ is the third leading cause of global cancer-related deaths.^15^ The International Agency for Research on Cancer estimates that the global burden of CRC will increase by 56% between 2020 and 2040, to more than 3 000 000 new cases per year, with an estimated 1 600 000 deaths worldwide in 2040.^14^

Between 1990 and 2019, the global number of new CRC cases more than doubled, rising from approximately 842 098 to 2 170 000, while deaths increased from 518 126 to 1 090 000.^16^ The overall burden, measured in disability-adjusted life years (DALYs; a measure of overall disease burden, with 1 DALY representing the loss of the equivalent of 1 year of good health), also doubled from 12,400,000 to 24,300,000 during the same period.^16^ However, these increases are not uniformly distributed across regions.^16^

The socio-demographic index (SDI) is a composite metric that identifies where countries or other geographic areas lie on the spectrum of development based on per capita income, average years of schooling, and total fertility rate.^17^ Data from the GBD study has shown that the incidence and death rates of CRC have decreased in regions with a high SDI.^16^ This may be due to effective screening and treatment programs. In contrast, low and middle SDI countries have experienced significant increases in CRC incidence and deaths, with cases doubling or more in 16 out of 21 world regions, particularly in Asia and Latin America.^16^

While overall CRC incidence and mortality are declining in high SDI regions, there is a contrasting, notable and concerning trend of substantial rise in CRC burden among individuals under 50 years of age—referred to as young-onset colorectal cancer (YOCRC).^16,18^ Gu et al^19^ showed that the incidence, mortality, and DALY rates of young-onset CRC (YOCRC) are increasing, particularly in high SDI regions.

YOCRC contrasts with late-onset colorectal cancer (LOCRC), which occurs in individuals aged 50 years and older and has shown stable or declining rates.^16^ The inconsistencies in incidence based on age suggest an impending epidemiological shift in CRC burden. The growing burden of YOCRC underscores the need for vigilance among researchers, clinicians, and policymakers. Therefore, this study aimed to (1) forecast the trends of incidence, death, and DALY rates of YOCRC and late-onset CRC (LOCRC) using data from the GBD Global Health Data Exchange (GHDx) (https://ghdx.healthdata.org/gbd-2019) and GLOBOCAN Cancer Over Time (https://gco.iarc.fr/overtime/en) tools, and (2) determine the approximate year when the incidence, death, and DALY rates of YOCRC and LOCRC will become equal.

## Materials and Methods

### Data Sources

We utilized data from the GBD study^20^ and GLOBOCAN database^21^ to obtain comprehensive estimates of CRC incidence, death, and DALY rates. The GBD provides systematic scientific evaluations of comparative risks and health loss due to diseases globally and has been extensively used in prior studies to assess CRC burden.^16^ GLOBOCAN offers the most recent estimates of cancer incidence and deaths worldwide, enhancing the robustness of our analysis.^21^

The GHDx provided incidence, death, and DALY rates from 1990 to 2019. The Cancer Over Time provided incidence and death data from 1943 to 2018. Considering that forecasting trends for every high SDI country will be cumbersome, we chose to forecast the trends for one high SDI country per continent to ensure wide geographic representation. For the specific country to be chosen from each continent, we chose to forecast the trends for the country with the highest SDI in that continent. Consequently, we chose Singapore, Australia, and Switzerland for Asia, Oceania, and Europe, respectively (there are no high SDI countries in Africa and South America). However, for North America, we chose the USA over Canada. This is because, while Canada has a slightly higher SDI than USA, the inclusion of the USA allows for better periodic updates of the forecasting models, particularly given its recent policy change in lowering the recommended CRC screening age from 50 to 45 years in response to a rising incidence among younger adults.^22^ Including the USA in the study allows us to analyze the impact of this policy change on CRC incidence, DALY, and mortality rates across the young- and late-onset groups in the next update of the forecasts. This could provide valuable insights into the effectiveness of earlier screening.

The detailed methodology for data extraction from GHDx and GLOBOCAN is provided in Supplemental methods 1.

### Age Group Definitions

We defined the 15-49- and 50-74-year age groups as the young- and late-onset groups, respectively, in this study. This was done to replicate a proven methodology with the use of a similar classification.^23^ Moreover, aggregate data for these age groups are readily available from the GBD study and GLOBOCAN through the GHDx and Cancer Over Time tools, respectively. The lower limit of 15 years for YOCRC is consistent with the GBD framework, which defines young-onset cancers as those occurring from age 15 onwards.^24^ The upper limit of 49 years aligns with global CRC screening guidelines, where screening typically begins at age 50 years. Similarly, the 50-74-year range for LOCRC corresponds to standard clinical practice for CRC screening, which generally starts at age 50 years and ceases at age 75 years.

### Data Forecasting and Evaluation

For a given forecast, we selected the longer time series data from either GBD or GLOBOCAN, depending on availability. The small dataset, consisting of annual observations for 30 years (in case of GBD) or a maximum of 75 years (in case of GLOBOCAN), presented limitations for advanced models like Neural Networks like Multilayer Perceptron and Long Short Term Memory Network, or complex ML models like XGBoost and Random Forest, which require substantial data for effective performance and are prone to high variance with limited samples (i.e., due to their complexity they start learning the noise from the data, instead of just the trend).^13^ To address these limitations (i.e., to minimize overfitting), we employed an ensemble of three simple forecasting methods, which are well-suited for handling short time series data^12^: SLR to capture linear trends, ExpSmoothing to emphasize recent data for short-term patterns, and ARIMA for modelling autocorrelation and non-stationarity to handle time series data. This approach allows us to address both the long-term patterns and the recent demographic shifts, providing a more robust and sensitive analysis of the increasing burden among younger populations.

We employed non-weighted averaging to ensemble the forecasts due to the limited size of our dataset and the need to minimize overfitting. With a maximum of 75 annual observations, estimating reliable weights for each model would be statistically challenging and could lead to overfitting. Non-weighted averaging avoids this issue by treating each model’s forecast equally. This approach leverages the complementary strengths of each model without bias toward any single one. Non-weighted averaging often performs robustly,^25^ while reducing computational complexity. Therefore, non-weighted averaging was deemed suitable for our datasets.

**Figure 1.**
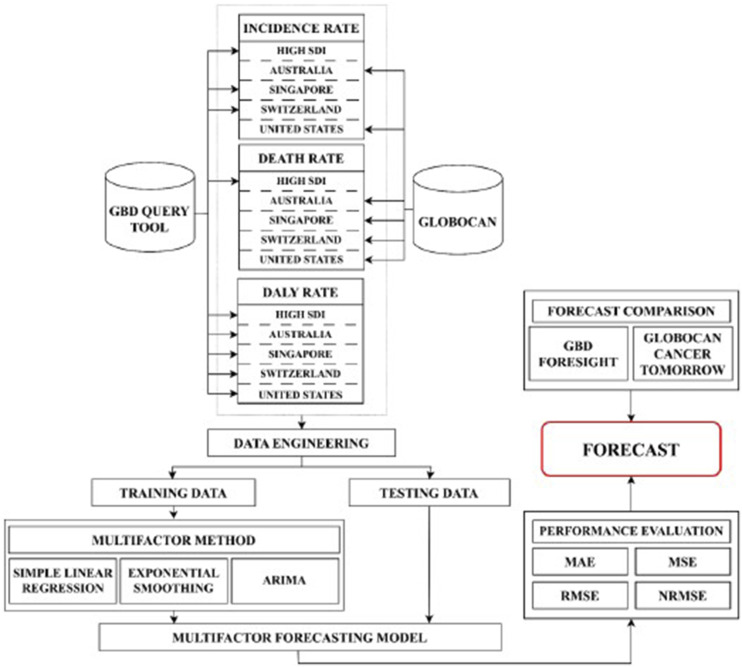
Forecast workflow. Arima: autoregressive integrated moving average; MAE: mean absolute error; RMSE: root mean squared error; NRMSE, normalised root mean squared error.

The workflow of the forecast is given in Figure 1. The detailed description of the models used is provided in Supplemental methods 2. The accuracy of the forecast was evaluated using the MAE, MSE, RMSE, and NRMSE (see Supplemental methods 3).

## Results

Across high SDI regions and the four high-SDI countries evaluated (Australia, Singapore, Switzerland, and the USA), YOCRC incidence generally increased, while LOCRC incidence declined. Consistent declines in LOCRC-related deaths and DALYs were observed, whereas YOCRC-related deaths and DALYs exhibited smaller decreases or varied trends depending on the specific country and sex.

### Trends in Incidence Rates

**Figure 2.**
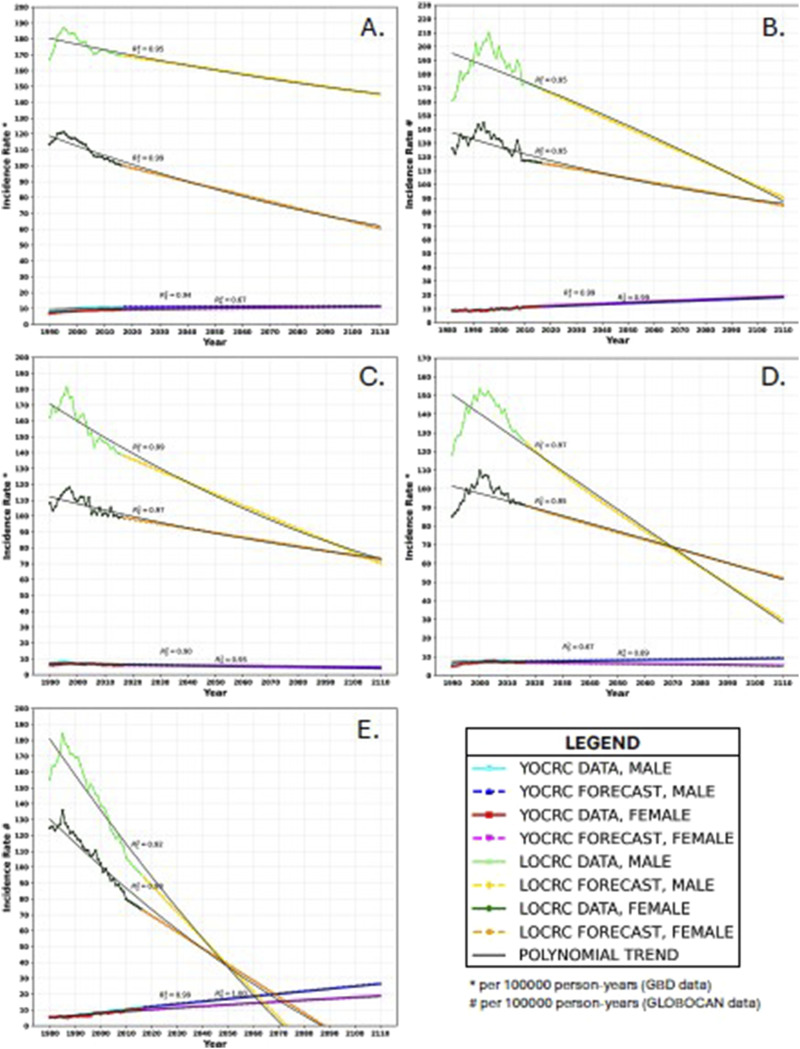
Trends in incidence rates in (A): the high socio-demographic index region, (B): Australia, (C): Singapore, (D): Switzerland, and (E): USA. The *R*^2^ values represent the trendline’s fit with the data and forecast. R_1_^2^ pertains to the incidence rate of late-onset colorectal cancer in males, R_2_^2^ pertains to the incidence rate of late-onset colorectal cancer in females, R_3_^2^ pertains to the incidence rate of young-onset colorectal cancer in males, and R_4_^2^ pertains to the incidence rate of young-onset colorectal cancer in females. All other symbols and colours are explained in the legend.

YOCRC incidence is increasing, while that of LOCRC is declining in high SDI regions and in all the countries evaluated. Figure 2 shows the current and projected trends in the incidence rates of YOCRC and LOCRC and Supplemental Table 1 shows the average rates of change (AROCs). Supplemental Table 2 shows the incidence rate data and forecasts.

In high SDI regions, the deceleration in the decrease of LOCRC in males is projected to be less prominent than that in females. Conversely, the rates of change in YOCRC incidence appear to be comparable between the two sexes. Our forecasts suggest that within the next 100 years, the incidence rates of YOCRC and LOCRC will not converge in either males or females within the high SDI regions.

In Australia, the incidence of LOCRC in females is expected to surpass that in males by 2068. Over the next 100 years, a convergence in the incidence rates of YOCRC and LOCRC is not expected for either sex.

In Singapore, the equalization of LOCRC incidence rates in males and females is anticipated by 2104. Nevertheless, within the next 100 years, there is no projected convergence in the incidence rates of YOCRC and LOCRC.

Switzerland is witnessing a decline in LOCRC incidence for both sexes, while YOCRC is expected to remain stable with slight increments. By 2069, the incidence of LOCRC in females is expected to surpass that in males. Over the next 100 years, a convergence in the incidence rates of YOCRC and LOCRC is not expected for either males or females.

In the USA, the rate of incidence of LOCRC in females is set to overtake that in males by 2050. The acceleration in the increase of YOCRC in males is projected to be more prominent than that in females. The incidence rates of YOCRC and LOCRC are set to equalize by 2073 in females and 2061 in males.

### Trends in Deaths Rates

**Figure 3.**
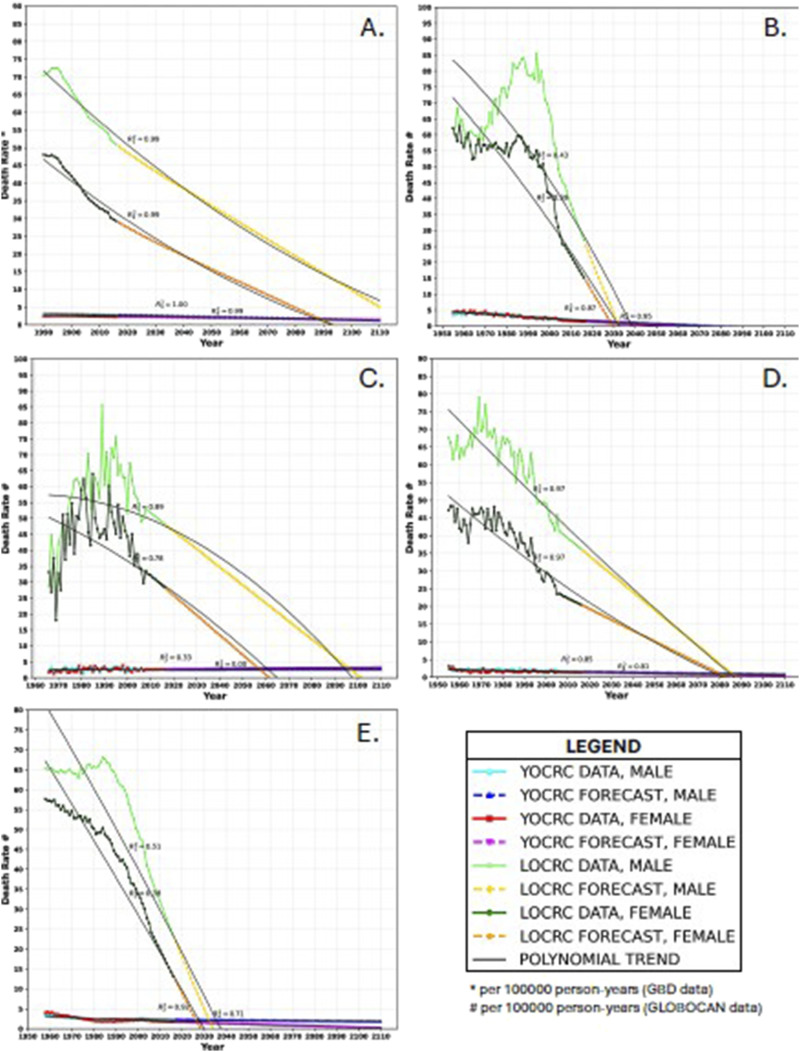
Trends in death rates in (A): the high socio-demographic index region, (B): Australia, (C): Singapore, (D): Switzerland, and (E): USA. The *R*^2^ values represent the trendline’s fit with the data and forecast. R_1_^2^ pertains to the death rate of late-onset colorectal cancer in males, R_2_^2^ pertains to the death rate of late-onset colorectal cancer in females, R_3_^2^ pertains to the death rate of young-onset colorectal cancer in males, and R_4_^2^ pertains to the death rate of young-onset colorectal cancer in females. All other symbols and colours are explained in the legend.

LOCRC death rates are markedly declining in both males and females in high SDI regions and in all the countries evaluated. Simultaneously, there is a negligible decrease in YOCRC death rates among both sexes in high SDI regions and Australia and Switzerland. In Singapore, the YOCRC death rates are increasing among both sexes, while in the USA, the YOCRC death rates are decreasing in females and are fairly stable with slight decreases in males. Figure 3 shows the current and projected trends in YOCRC and LOCRC death rates and Supplemental Table 3 shows the AROCs. Supplemental Table 4 shows the death rate data and forecasts.

In high SDI regions, the death rates of YOCRC and LOCRC are expected to equalize in females and males by the years 2089 and 2118, respectively. Predictions for Australia suggest that YOCRC and LOCRC death rates will intersect in males and females by the years 2031 and 2029, respectively. In Singapore, our projections suggest that YOCRC and LOCRC death rates will converge in males and females by the years 2097 and 2057, respectively. Our forecasts for Switzerland indicate that YOCRC and LOCRC death rates will equalize in males and females by the years 2085 and 2081, respectively. Projections for the USA suggest that YOCRC and LOCRC death rates will converge in males and females by the years 2032 and 2027, respectively.

### Trends in DALY Rates

**Figure 4.**
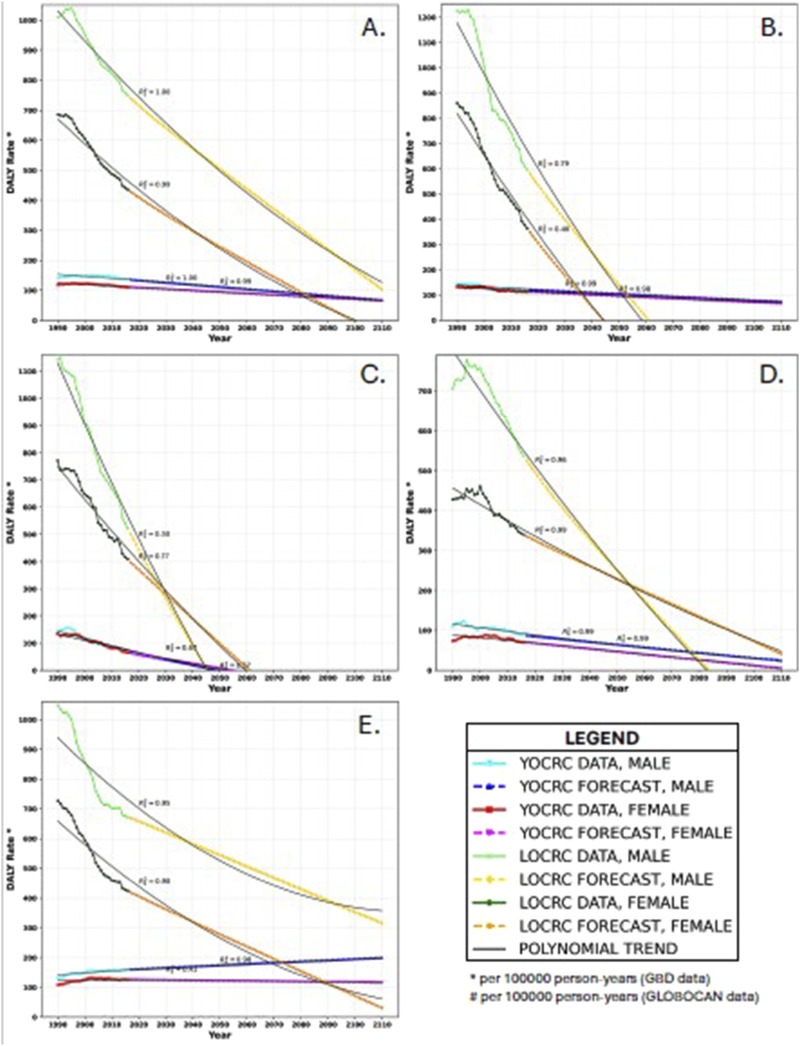
Trends in disability-adjusted life year (DALY) rates in (A): the high socio-demographic index region, (B): Australia, (C): Singapore, (D): Switzerland, and (E): USA. The *R*^2^ values represent the trendline’s fit with the data and forecast. R_1_^2^ pertains to the DALY rate of late-onset colorectal cancer in males, R_2_^2^ pertains to the DALY rate of late-onset colorectal cancer in females, R_3_^2^ pertains to the DALY rate of young-onset colorectal cancer in males, and R_4_^2^ pertains to the DALY rate of young-onset colorectal cancer in females. All other symbols and colours are explained in the legend.

LOCRC DALY rates are declining sharply in both males and females in high SDI regions and in all the countries evaluated. Simultaneously, YOCRC DALY rates are gradually declining in both males and females in high SDI regions and in all the countries evaluated except USA. In USA, the YOCRC DALY rates are increasing in both males and females. Figure 4 shows the current and projected trends of YOCRC and LOCRC DALY rates and Supplemental Table 5 shows the AROCs. Supplemental Table 6 shows the DALY rate data and forecasts.

In high SDI regions, our projections indicate that the DALY rates for both YOCRC and LOCRC will intersect for females and males by 2084 and 2116, respectively. For Australia, the forecast suggests convergence in DALY rates for YOCRC and LOCRC in males and females by 2053 and 2037, respectively. In Singapore, the DALY rate for LOCRC in females is anticipated to surpass that in males by 2028. The DALY rates for YOCRC and LOCRC in males are expected to converge in 2045, while equalization is not projected for females within the next 100 years. In Switzerland, the forecast predicts that the DALY rate for LOCRC in females will exceed that in males by 2056. Intersection is expected in DALY rates for YOCRC and LOCRC in males by 2077, with no projected convergence in females within the next 100 years. For the USA, DALY rate convergence for YOCRC and LOCRC in females is projected by 2089. In males, equalization is not expected within the next 100 years.

## Discussion

In this study, we developed a novel multifactor forecasting model capable of providing reliable long-term forecasts of CRC burden using short time-series data. Our model successfully projected incidence, death, and DALY rates for YOCRC and LOCRC over a 90-year horizon. Notably, our findings indicate a significant rise in YOCRC incidence and death rates in high SDI regions, consistent with previous results,^18,26-28^ with a projected convergence of YOCRC and LOCRC rates in the coming decades. Additionally, contrary to the prevailing condition where it has been determined that males experience a higher incidence of YOCRC^18,29^ and LOCRC,^30^ our study suggests that in the coming years, it is likely that females may experience a higher YOCRC and LOCRC burden. These results fulfill our study objectives to develop an efficient forecasting model and to identify trends in CRC burden in the high SDI region.

Early detection of CRC is crucial for better patient outcomes. Notably, the landscape of CRC is evolving, with an increase in YOCRC^18^ and a decrease in LOCRC.^31^ Hence, accurately understanding the evolution of CRC and responding appropriately by developing forecasts for incidence, death, and DALY rates are vital for resource allocation and effective management of CRC.

Prior to this study, we conducted a systematic review of CRC burden forecasting studies which revealed predictions up to 2019 (we excluded the forecasts from GBD and GLOBOCAN as they met our exclusion criteria of not reporting their performance metrics).^11^ We found that no study, including the studies we excluded from the review, forecasted DALYs and that the longest forecast length among the included studies was 16 years.^11^ The longest forecast length in the extant literature is 28 years (until 2050), achieved by GLOBOCAN’s Cancer Tomorrow.^6^ Our study exceeded this, offering 90-year forecasts with strong performance. Cancer Tomorrow predicts incidence and death counts rather than rates,^6^ potentially limiting its utility when predicting demographic shifts and inflection points. GBD’s Foresight (until 2040) predicts an annual percentage of change of −0.38% and −0.77% for YOCRC and LOCRC deaths, respectively, from 1990 to 2040 in high-income regions.^5^ A direct comparison of these forecasts with ours is not possible as the age groups were defined differently within Foresight. Moreover, Foresight lacked forecasting for high SDI regions and incidence and DALY rates. Additionally, the data used to predict death rate trends are not comparable due to Foresight using data reported as deaths per 100 000,^5^ while the GBD used data reported as deaths per 100 000 person–years.^32^

The present study is a much-needed contribution to the field of disease forecasting, particularly in relation to CRC. A distinctive feature of our study lies in the comprehensive analysis of CRC trends, encompassing not only overall incidence and death rates but also DALY rates. The identification of forecasted inflection points, particularly in death rates, equips our forecast with the capacity to predict epidemiological shifts accurately and meaningfully. Additionally, by extending our forecast to 90 years, our research provides a long-term perspective, which is indispensable for healthcare planning and resource allocation, allowing policymakers to anticipate and prepare for changes in disease burden over extended periods. Furthermore, our models outperformed the previous forecast models^9^ (see Supplemental Tables 7 and 8 for the performance metrics of the models described herein).

Similar to the disease forecasting models developed during COVID-19 that proved valuable in the real-time monitoring of the impact of interventions,^33^ the ML pipeline developed in this study can be a valuable tool for real-time monitoring of the impact of interventions targeting specific groups for CRC prevention. Considering that there is usually a lag time between the implementation of an intervention and its observable impact on disease burden or other health outcomes, an extended forecast, particularly in the context of CRC deaths, can provide a baseline and once interventions, such as targeted screening programs or awareness campaigns, are introduced, the pipeline can continuously analyse incoming data, monitoring changes in the inflection points, providing a quantifiable impact of the interventions on different groups, including age, sex, and geographical regions over time. This also allows for quick adjustments and fine-tuning of strategies based on emerging trends and the effectiveness of ongoing interventions. The pipeline also supports a comparative analysis between groups or regions with implemented interventions and those without. This comparative approach helps assess the relative effectiveness of specific interventions and informs decisions about scaling up successful strategies.

### Interpretation of National Trends

#### Australia

Despite the national screening program recommending a colonoscopy within 30, to a maximum of 120, days after a positive screening test in Australia,^34^ data from 2014 to 2021 demonstrate an average wait time of 145.75 ± 5.04 days nationwide.^35^ Bowel Cancer Australia is urging the government to lower the screening age.^36^ However, this change may worsen waiting times. While the forecast for Australia indicates convergence in death rates for LOCRC and YOCRC in males and females by 2031 and 2029, respectively, and in DALY rates by 2053 for males and 2037 for females, the extended time between a positive test and colonoscopy could impact outcomes.^37^ Urgently addressing this gap in wait times is crucial to ensure the screening program’s efficacy and align preventive measures with the evolving landscape of CRC in Australia, thereby contributing to better CRC prevention and management in Australia.

#### Singapore

This study projects a relatively favourable outlook for Singapore, with the anticipation of LOCRC and YOCRC incidence rates converging in males and females by 2104 and 2360, respectively. The declining LOCRC death rates in both sexes indicate effective management. However, the increasing YOCRC death rates pose a concern. With a national bowel cancer screening program for individuals aged over 50 years^38^ and an average waiting time of only 38 days for a colonoscopy in a public hospital,^39^ Singapore’s health system has the capacity to widen the scope of its screening program by lowering the age for eligibility of screening to address the rising trend in YOCRC. The anticipated convergence of death rates by 2057 in females and 2203 in males emphasizes the need for targeted interventions and resource planning to minimize the sex disparity.

#### Switzerland

As of 2023, 10 Cantons in Switzerland did not organize CRC screening programs,^40^ and in those that offered the screening programs, the age groups included in the programs were 50-69-year-olds.^41^ However, only 8.3% of the eligible population underwent a screening test in 2014, 8.9% in 2016, and 9.2% in 2018.^42^ Despite the absence of a comprehensive CRC screening program and low screening uptake, the present study shows a favourable CRC burden forecast compared to the other countries evaluated. This suggests that there exist factors beyond screening alone that may contribute to positive outcomes. These may include a healthy diet,^43^ increased physical activity,^44^ and other positive health-related behaviours and health system-related factors.^45,46^ The findings underscore the importance of a comprehensive approach to CRC prevention and management.

#### USA

In USA, the national bowel cancer screening program recommends a timely follow-up colonoscopy after a positive screening test for individuals aged over 50 years. Data shows that the mean waiting time for a diagnostic colonoscopy following a positive screening test was 54 days in 2016.^47^ However, disparities exist owing to differences in access to health insurance.^48-50^ Based on the findings of this study, the USA appears to be the least affected by sex disparity in terms of CRC burden. However, it is set to experience a substantial increase in the burden of YOCRC, particularly amongst males. The American Cancer Society and The United States Preventive Services Task Force guidelines now recommend a lower age for CRC screening.^22,51^ Monitoring the disease burden in the USA, henceforth, will provide insights into the effectiveness of lowering the CRC screening age. To effectively manage the growing public health threat of YOCRC and the overall CRC burden worldwide, it is imperative that experience with this intervention is shared widely in a timely manner.

The findings of this study underscore the imperative for global collaboration in addressing the challenges posed by the evolving demographics of CRC. Global collaboration is crucial for sharing best practices and lessons learned. Collaborative efforts can extend to the establishment of standardized screening protocols, harmonized data collection methodologies, and joint research initiatives. International organizations can foster collaboration by facilitating forums for knowledge exchange, supporting joint research endeavours, and advocating for increased attention and resources directed toward YOCRC prevention and control on the global health agenda.

### Implications for Health Policy and Practice

The projected increase in YOCRC burden in high SDI regions has significant implications for health policy and practice. Our predictive model serves as a critical tool for informing strategies in several key areas:

#### Resource Allocation and Healthcare Planning

Our forecasts enable health authorities to anticipate future demands on healthcare systems. Policymakers can utilize these insights to allocate resources effectively, ensuring that healthcare infrastructure and workforce capacity are scaled appropriately. This includes investing in diagnostic and treatment facilities, expanding screening services, and training healthcare professionals specialized in general surgery, oncology, and gastroenterology to manage the anticipated increase in YOCRC cases.

#### Targeted Cancer Screening Programs

While lowering the universal screening age may lead to unsustainable increases in healthcare professionals’ workloads, our findings underscore the need for targeted screening strategies focused on high-risk groups. Screening programs can be optimized by incorporating risk factors such as family history and genetic predispositions (e.g., individuals with Lynch syndrome can be prioritised), lifestyle and environmental factors (e.g., screening criteria can include individuals with significant lifestyle risks like obesity, smoking, and poor diet), and specific high-risk population subgroups demonstrating higher YOCRC incidence (e.g., the Indigenous communities of Australia, as suggested by Lew et al (2022),^52^ could be prioritized for tailored screening programs). This targeted approach enhances cost-effectiveness, optimizes resource utilization, and potentially improves early detection rates among those most at risk.

#### Holistic Care Planning for YOCRC Patients

Younger CRC patients often face unique challenges, including psychological impacts, fertility concerns, and socioeconomic burdens. Healthcare systems should develop comprehensive care models that address these needs, improving patient outcomes and quality of life. This includes providing mental health services, fertility preservation options, and support for managing financial and familial responsibilities.

#### Public Health Campaigns and Education

Our findings highlight the importance of increasing awareness of CRC symptoms among younger populations. Education campaigns that encourage timely medical consultation for early symptoms must be designed to facilitate earlier diagnoses and better outcomes. It is important to communicate that while CRC has historically been more prevalent in individuals over 50 years old, there is a growing incidence among younger people. Raising awareness about this shift is crucial for both the public and healthcare providers to ensure early detection and prompt treatment.

#### Research Investment to Understand Underlying Causes

Addressing the increasing YOCRC trend requires a deeper understanding of its underlying causes. Policymakers must allocate funding toward research initiatives exploring genetic, environmental, and lifestyle factors contributing to YOCRC. Such research can inform the development of targeted prevention strategies and public health interventions aimed at reducing incidence rates.

### Limitations and Strengths

Our study presents several limitations. First, similar to all forecasting studies, our reliance on historical data assumes that future trends in CRC burden will follow past patterns. This assumption may not hold true if there are unforeseen factors such as new interventions, changes in healthcare policies, shifts in risk factors, or significant public health events like pandemics. To address this, we plan to periodically update our forecasts to incorporate the most recent data and account for significant changes, such as the recent lowering of the CRC screening age from 50 to 45 years in USA.

Second, the small size of the cancer burden datasets, comprising annual observations over 30-75 years, limits the complexity of models that can be employed without risking overfitting. While our use of an ensemble of SLR, ExpSmoothing, and ARIMA models is appropriate for handling short time series data, our method may not capture complex nonlinear relationships inherent in epidemiological trends.

Third, SLR, ExpSmoothing, and ARIMA make specific assumptions, such as linearity, stationarity, and the absence of significant autocorrelation beyond the model order, which may not fully hold in real-world data. Additionally, due to data limitations, traditional cross-validation techniques were not feasible (see Supplemental Methods 3). We relied on out-of-sample testing for model validation, but this approach may not fully capture the model’s generalizability.

Fourth, the specific findings of this study are applicable only to the high SDI region and the countries analysed. Although our model is adaptable and allows for broader application, it is important to recognize that forecasts need to be tailored to specific populations due to differences in epidemiological patterns, healthcare systems, and risk factor profiles.

Lastly, the age cutoffs chosen in this study could overlook certain age groups that may have relevant characteristics, such as those just outside the specified age ranges. However, these age-group boundaries were chosen to best align with current clinical and epidemiological standards, given the lack of a universally agreed-upon definition for YOCRC. Thus, we believe that our approach remains a reasonable starting point for further investigation, while recognizing that age classifications may evolve as the field progresses.

Despite the significant limitations, our study presents several significant strengths that advance not only the field of disease burden forecasting but also the broader discipline of forecasting methodologies. A primary strength is our successful development and validation of a novel multifactor forecasting model capable of handling short time series data to produce reliable long-term forecasts. Short time series data pose a common challenge in many fields, including epidemiology, economics, environmental science, and social sciences, where historical data may be limited due to factors such as recent emergence of phenomena, changes in measurement techniques, or inconsistent data collection practices. Using our ensemble model, we have attempted to overcome these limitations and generate reliable forecasts extending up to 90 years.

Another key strength is the identification of specific inflection points in the trends of CRC incidence, death, and DALY rates. This capability is crucial for anticipating epidemiological shifts and enables policymakers to implement timely interventions, enhancing the relevance of our study for healthcare planning.

The adaptability of our forecasting model for real-time monitoring and evaluation of public health interventions is another notable strength. The model’s design allows for periodic updates with new data, enabling the assessment of the immediate impact of interventions or sudden changes in risk factors, such as modifications in screening guidelines or shifts in population behaviors. This feature enhances the model’s utility in evaluating the effectiveness of policies and can inform adjustments to strategies based on emerging trends.

The simplicity and accessibility of our forecasting model are also significant advantages. By utilizing simple methods that require fewer computational resources, the model remains accessible to a wide range of researchers and practitioners who may not have expertise in complex ML techniques or lack access to advanced computational resources. This simplicity facilitates easier implementation and adoption in various healthcare settings without the need for advanced technical skills.

Furthermore, our study contributes to global health knowledge by focusing on high SDI regions across different continents, adding to the global understanding of CRC dynamics. It highlights regional differences and commonalities in CRC trends, emphasizing the need for international collaboration in addressing this public health challenge.

Finally, our forecasts are based on well-known robust datasets. This strengthens the credibility of the findings and provides a solid foundation for future research. We address a critical gap in the literature by providing long-term forecasts extending up to 90 years, including DALY rates, which were previously unaddressed.

### Directions for Future Research

This study represents an innovative effort in forecasting CRC burden, providing valuable insights for policymakers and healthcare professionals. We plan to periodically update our forecasts to incorporate the most recent data. Our next update will enable us to assess the impact of earlier screening on CRC burden in USA and provide evidence on its effectiveness to inform policy decisions in other countries. Moving forward, we aim to forecast the CRC burden in the remaining SDI regions. Additionally, we will broaden our scope to include the forecasting of other gastrointestinal cancers. An interface to enable other researchers to use our ML pipeline to forecast any disease burden using annual data is in development. While the age classifications used in this study reflect current global standards and practices and provide a reasonable framework for analysis, it is important to note that the definition of YOCRC remains an evolving area of research. The selection of the specific age boundaries for YOCRC and LOCRC is subject to ongoing debate in the literature, and future studies may refine these cutoffs as more data on the biological, epidemiological, and clinical characteristics of YOCRC become available. A better understanding of the timing and effectiveness of current treatments for cancer in the young are the need of the hour as much of our understanding of the treatment of cancers comes from people aged 50-70 years.^53^

## Conclusions

The landscape of colorectal cancer is shifting, with a significant rise in young-onset cases anticipated in high SDI regions over the coming decades. By integrating robust predictive modeling with informed health policies, we can optimize resource allocation, improve patient outcomes, and ultimately reduce the impact of CRC on society. Resources should be allocated to expand and target screening programs toward younger, high-risk populations, particularly focusing on females. Implementing public health campaigns to raise awareness of CRC symptoms among younger age groups can facilitate early detection of YOCRC. Investing in research to understand the underlying causes of the rising YOCRC trend is essential. By proactively adjusting screening guidelines, enhancing patient education, and prioritizing research funding, policymakers can mitigate the impact of the projected increase in YOCRC burden and improve health outcomes.

## Supplemental Material

Supplemental Material - Evolving Dynamics of Colorectal Cancer in High Socio-Demographic Regions Supplemental Material for Evolving Dynamics of Colorectal Cancer in High Socio-Demographic Regions by Laalithya Konduru, Simranjeet Singh Dahia, Claudia Szabo, and Savio G. Barreto in Cancer Control

## Appendix

### List of Abbreviations

AAPC: Average annual percentage change
AROC: Average rate of change
ARIMA: AutoRegressive integrated moving average
CRC: ColoRectal cancer
DALY: Disability-adjusted life year
ExpSmoothing: Exponential smoothing
GBD: Global burden of disease
GLOBOCAN: Global cancer observatory
ML: Machine learning
MSE: Mean squared error
MAE: Mean absolute error
NRMSE: Normalized RMSE
RMSE: Root MSE
SLR: Simple logistic regression
SDI: Socio-demographic index
YOCRC: Young-onset CRC
LOCRC: Late-onset CRC.
