# Supplementary material for: Evolving Dynamics of Colorectal Cancer in High Socio-Demographic Regions
Source: Cancer Control. 2025 Feb 17;32:10732748251321672. doi: 10.1177/10732748251321672 (PMC11833813; doi:10.1177/10732748251321672)
Supplement: Supplemental Material - Evolving Dynamics of Colorectal Cancer in High Socio-Demographic Regions [file sj-pdf-1-ccx-10.1177_10732748251321672.pdf]

Supplemental Materials

Evolving Dynamics of Colorectal Cancer in High Socio-demographic Regions

Table of contents

S.No	Content	Page Number
Supplemental methods 1	Data Extraction	2
Supplemental methods 2	The configuration of the methodologies used and the pseudocode of the novel multifactor model	3
Supplemental methods 3	Performance evaluation method	4
Supplemental Table 1	The average annual rates of change in incidence rates	5
Supplemental Table 2	Incidence rate data and forecasts	6
Supplemental Table 3	The average annual rates of change in death rates	11
Supplemental Table 4	Death rate data and forecasts	12
Supplemental Table 5	The average annual rates of change in DALY rates	18
Supplemental Table 6	DALY rate data and forecasts	19
Supplemental Table 7	Performance metrics for each YOCRC forecast	25
Supplemental Table 8	Performance metrics for each LOCRC forecast	26
Supplemental References	Studies referred to in the Supplemental material	27

Supplemental methods 1. Data Extraction

For data extraction from GHDx, we used “Incidence”, “Deaths”, and “DALYs” as the “Measure”; “Rate” as the “Metric”; “Male” and “Female” as “Sex”; “15–49 years” and “50–74 years” as “Age”; and “Australia”, “High SDI”, “Singapore”, “Switzerland” and “USA”, as “Location” for the years 1990–2019. The rates were per 100,000 person–years.

For data extraction from Cancer Over Time, we used “Population” as “Display by”; “Crude Rate” as “Statistics”; “Mortality” as “Measures”; “Males” and “Females” as “Sexes”; “Colorectum” as “Cancer Sites”; “Australia”, “Singapore”, “Switzerland” and “USA” as “Populations”; “15–49” and “50–74” as “Age groups”; and “1943–2018” as years. The Crude rate was defined as a rate per 100,000 persons per year.

Supplemental methods 2. The configuration of the methodologies used and the pseudocode of the novel multifactor model

SLR

A detailed description of the features, strengths, and limitations of SLR is given elsewhere.[1]

In this study, we employed SLR using Scikit-learn's LinearRegression library, and matplotlib.pyplot was used for data visualization.

ExpSmoothing

A detailed description of the features, strengths, and limitations of ExpSmoothing is given elsewhere.[1]

We used the "ExponentialSmoothing" function by the "statsmodels" library, which implements the Holt−Winters ExpSmoothing Method assigning different weights to historical data points for forecasting future values.[1] The model was configured to incorporate an additive trend and no seasonal component, meaning the trend component would be added without considering recurring seasonal patterns.

ARIMA

A detailed description of the features, strengths, and limitations of ARIMA is given elsewhere.[1]

In our model, we used the "ARIMA" function by the "statsmodels" library which provides the basic interface for ARIMA-type models. We configured the order of ARIMA parameters as (p=2, d=1, q=1), after evaluating different values of p,d, and q to generate forecasts. In this configuration, p=2 means that the model considers the two most recent time steps for prediction based on their autoregressive relationships, d=1 means the data is differenced (subtracting the current observation from a previous one) once to make it stationary, and q=1 means that the model considers the most recent forecast error.

The Final Forecast Model Used In This Study

We combined SLR, ExpSmoothing, and ARIMA forecasts using the mean aggregation method[2] to produce the final forecast. This approach enhances prediction accuracy and reliability by leveraging the strengths of diverse models, capturing various data aspects for a more robust multifactor forecasting method. The configurations of the individual component models of our multifactor model ensure an effective representation of the observed patterns and dynamics, making it suitable for forecasting future observations based on historical data

Pseudocode

```
def multifactor_method(cancer_burden_data, forecast_years):
    "A method to forecast colorectal cancer burden using three different forecasting models.
    Parameters:
    - cancer_burden_data: dataset containing the historical data of colorectal cancer burden.
    - forecast_years: number of years into the future to forecast.
    Returns:
    - forecast: an array or list of predicted values representing the forecasted cancer burden.
    "
    # Forecast using Simple Linear Regression
    forecast_slr = linear_regression(cancer_burden_data, forecast_years)
    # Forecast using Exponential Smoothing
    forecast_exponential_smoothing = exponential_smoothing(cancer_burden_data, forecast_years)
    # Forecast using ARIMA
    forecast_arima = arima(cancer_burden_data, forecast_years)
    # Combine forecasts by mean aggregation method2
    forecast = (forecast_slr + forecast_exponential_smoothing + forecast_arima) / 3
    return forecast
```

Supplemental methods 3. Performance evaluation method

Due to the limited length of the time series data, traditional cross-validation techniques were not feasible. Cross-validation methods require partitioning the dataset into multiple subsets to iteratively train and validate the model. With limited data in our case, this would have resulted in training and validation sets that are too small to produce reliable and generalizable estimates, by reducing the effective sample size in each training iteration, increasing the risk of overfitting and estimation variance. Instead, we used out-of-sample testing by dividing the data into training and testing sets and calculating the performance metrics of our forecasts.

The following performance metrics were calculated using the testing data and forecasted values:

Mean Absolute Error (MAE)

MAE represents the average absolute difference between the predicted and actual values. Mathematically,

$$MAE = \frac{1}{N}\sum_{i=1}^{N}|Predicted_i - Actual_i|$$

Mean Squared Error (MSE)

MSE represents the average squared differences between predicted and actual values. Squaring emphasizes larger errors, making MSE sensitive to outliers. Mathematically,

$$MSE = \frac{1}{N}\sum_{i=1}^{N}(Predicted_i - Actual_i)^t$$

Root Mean Squared Error (RMSE)

RMSE represents the square root of MSE, quantifying error magnitude in the original units of the data, making it easier to communicate the practical significance of the forecasting model's accuracy. Mathematically,

$$RMSE = \sqrt{MSE} = \sqrt{\frac{\sum_{i=1}^{N}(Predicted_i - Actual_i)^t}{N}}$$

Normalized Root Mean Squared Error (NRMSE)

NRMSE represents RMSE normalized by the original data range, allowing a comparison of the model's performance relative to the scale of the data. It is a useful metric for assessing accuracy across different datasets with varying ranges. Mathematically,

$$NRMSE = \frac{RMSE}{mean} = \frac{\sqrt{\frac{\sum_{i=1}^{N}(Predicted_i - Actual_i)^t}{N}}}{|Actual_{max} - Actual_{min}|}$$

For all of these metrics, a lower value indicates better relative performance.

We experimented with various test–train ratios and compared the above performance metrics. As per the Pareto Principle,[3] we selected 20:80 as the optimal test–train ratio as it minimized the overall NRMSE across all forecasts.

Supplemental Table 1. The average annual rates of change in incidence rates

Region	Group	Gender	Average AROC of available data	Average AROC of available data and 30-year forecast	Average AROC of available data and 50-year forecast	Average AROC of available data and 90-year forecast
High SDI	YOCRC	Male	1·19%	0·58%	0·45%	0·31%
		Female	1·09%	0·63%	0·52%	0·41%
	LOCRC	Male	0·05%	-0·06%	-0·09%	-0·12%
		Female	-0·47%	-0·46%	-0·48%	-0·52%
Australia	YOCRC	Male	1·41%	1·02%	0·90%	0·75%
		Female	1·07%	0·84%	0·76%	0·66%
	LOCRC	Male	0·17%	-0·15%	-0·26%	-0·42%
		Female	-0·21%	-0·25%	-0·27%	-0·29%
Singapore	YOCRC	Male	-0·28%	-0·34%	-0·37%	-0·42%
		Female	-0·12%	-0·19%	-0·21%	-0·23%
	LOCRC	Male	-0·55%	-0·57%	-0·59%	-0·68%
		Female	-0·26%	-0·29%	-0·30%	-0·32%
Switzerland	YOCRC	Male	0·82%	0·51%	0·44%	0·36%
		Female	1·22%	0·42%	0·24%	0·06%
	LOCRC	Male	0·18%	-0·47%	-0·66%	-1·10%
		Female	0·25%	-0·14%	-0·25%	-0·39%
USA	YOCRC	Male	2·14%	1·69%	1·50%	1·25%
		Female	1·90%	1·42%	1·25%	1·03%
	LOCRC	Male	-1·34%	-1·89%	-3·18%	-7·82%
		Female	-1·42%	-1·61%	-2·02%	6·65%

(Abbreviations: AROC, Annual rate of change; LOCRC, Late-onset colorectal cancer; SDI, Socio-demographic index; YOCRC, Young-onset colorectal cancer)

Supplemental Table 2. Incidence rate data and forecasts

Year	High SDI				Australia				Singapore				Switzerland				United States			
	Young Onset CRC		Late Onset CRC		Young Onset CRC		Late Onset CRC		Young Onset CRC		Late Onset CRC		Young Onset CRC		Late Onset CRC		Young Onset CRC		Late Onset CRC	
	Male	Female	Male	Female	Male	Female	Male	Female	Male	Female	Male	Female	Male	Female	Male	Female	Male	Female	Male	Female
1980																	5.596	5.314	155.283	124.687
1981																	5.562	5.426	163.746	125.599
1982					7.769	8.830	160.860	126.178									5.749	5.232	163.832	123.198
1983					9.511	8.436	163.561	122.448									5.468	5.664	167.530	125.526
1984					8.254	8.686	171.188	128.320									5.886	6.047	172.727	127.122
1985					8.785	8.958	181.990	136.148									6.095	5.306	184.128	135.750
1986					8.782	8.809	176.042	133.586									5.933	5.291	178.057	127.735
1987					8.670	9.547	180.311	134.030									6.037	4.944	176.085	125.378
1988					8.898	8.113	180.825	128.761									5.870	5.383	171.357	121.298
1989					8.111	7.921	189.045	133.106									6.148	6.037	171.176	122.203
1990	8.061	6.885	167.131	113.607	8.613	8.892	186.401	134.239	7.102	6.209	162.116	108.171	6.282	4.938	118.309	85.246	6.425	6.110	169.489	120.049
1991	8.291	7.067	170.803	115.237	8.204	9.038	202.391	138.953	7.480	5.863	169.380	103.520	7.011	5.135	126.044	86.971	6.588	5.891	169.453	117.528
1992	8.572	7.235	175.526	116.766	8.813	9.159	193.629	143.525	7.723	6.154	165.453	106.143	7.232	5.410	128.375	88.661	7.231	6.249	164.553	116.662
1993	8.930	7.533	182.025	120.228	9.882	8.189	198.524	139.804	8.057	6.398	167.149	110.538	7.290	5.838	129.147	92.171	6.755	6.141	159.258	112.792
1994	9.115	7.659	184.070	120.474	8.740	8.670	203.254	144.740	8.478	6.462	173.408	112.994	7.748	6.215	135.380	93.760	6.531	6.145	154.955	110.833
1995	9.391	7.900	186.991	121.194	9.162	8.390	204.532	138.041	8.628	6.836	176.321	115.205	7.464	6.501	143.880	99.792	7.096	6.132	148.045	110.574
1996	9.542	8.019	185.767	119.624	9.711	8.850	210.034	138.223	8.658	6.977	181.222	116.813	7.226	6.331	140.437	97.529	7.491	6.320	152.331	106.925
1997	9.655	8.120	182.833	117.798	9.216	10.070	202.716	133.449	7.805	7.178	174.459	117.954	7.525	6.644	145.278	101.899	8.160	7.354	150.191	107.987
1998	9.790	8.192	183.410	117.208	9.602	9.118	195.100	135.939	7.600	7.272	175.099	115.104	7.521	6.764	149.897	100.602	8.424	7.723	145.992	110.381
1999	9.909	8.334	183.281	117.386	9.306	10.376	191.484	133.033	6.860	6.960	164.122	110.748	7.150	6.766	147.315	102.793	7.758	8.096	144.439	104.894
2000	10.112	8.451	180.223	116.225	10.565	9.761	200.103	132.966	6.967	6.740	159.290	109.501	7.825	7.059	153.678	109.652	8.856	7.305	138.600	102.285
2001	10.328	8.618	178.192	114.849	10.055	10.323	192.643	131.330	7.248	6.614	162.835	112.042	7.886	7.225	150.721	106.185	8.780	8.135	137.906	96.918
2002	10.530	8.715	177.609	113.966	9.404	10.091	189.510	126.312	7.552	6.410	164.361	112.114	8.051	7.701	149.989	107.335	8.692	8.344	135.297	98.917
2003	10.760	8.839	178.076	113.237	9.169	10.133	184.313	126.376	7.418	6.406	158.936	106.952	8.052	7.689	152.022	106.541	9.440	7.860	131.045	95.373
2004	10.767	8.833	174.204	110.449	9.796	9.809	187.167	124.001	6.974	7.033	151.126	111.716	8.358	7.569	149.612	100.871	9.557	8.282	123.471	91.853
2005	10.904	8.901	173.421	108.781	10.061	10.069	181.440	120.957	6.997	6.787	152.684	101.286	8.423	7.763	147.622	98.003	9.786	8.543	121.106	92.398
2006	10.935	8.871	170.331	106.381	10.341	10.739	182.297	125.001	6.841	6.944	143.170	103.287	8.087	7.807	144.507	99.950	10.048	9.116	117.909	88.668
2007	11.021	8.919	170.688	105.683	10.263	11.514	189.871	131.935	6.890	6.436	147.436	101.034	8.042	7.421	144.922	100.622	9.977	8.946	120.177	88.903

2008	11.215	9.133	172.192	105.885	10.584	10.173	186.672	125.757	7.099	6.428	149.677	105.248	8.178	7.049	140.356	97.421	10.283	8.498	115.393	86.297
2009	11.369	9.298	173.299	105.427	11.583	11.129	172.329	117.341	6.742	6.035	147.325	100.672	8.434	7.030	142.579	96.897	10.098	9.089	112.979	84.587
2010	11.314	9.218	172.884	104.225	11.241	11.379	174.681	117.211	6.626	6.024	146.452	101.914	8.121	7.429	137.404	97.241	10.793	9.486	105.735	79.818
2011	11.216	9.256	172.224	104.391	11.370	11.485	173.458	117.329	6.683	6.153	145.510	100.195	7.898	7.273	133.295	92.666	10.941	9.556	103.998	78.715
2012	11.057	9.125	171.061	102.947	11.448	11.548	172.923	117.132	6.350	6.440	142.360	105.291	7.635	7.260	130.691	94.036	11.104	9.651	101.973	77.665
2013	11.002	9.093	170.907	102.644	11.511	11.626	171.846	116.810	6.543	5.799	143.500	100.176	7.769	6.872	131.115	92.421	11.262	9.747	100.386	76.630
2014	11.229	9.277	170.185	101.342	11.585	11.699	171.197	116.470	6.498	5.911	140.133	100.250	7.680	6.962	128.643	92.553	11.421	9.840	98.541	75.604
2015	11.217	9.295	169.983	100.938	11.657	11.774	170.210	116.137	6.490	5.846	139.431	99.033	7.683	6.922	127.319	91.946	11.580	9.933	96.963	74.579
2016	11.204	9.292	169.546	100.337	11.729	11.848	169.490	115.809	6.462	5.881	138.695	99.624	7.706	6.886	125.840	91.412	11.739	10.026	95.192	73.556
2017	11.195	9.315	169.387	99.985	11.801	11.923	168.559	115.481	6.442	5.820	137.968	98.516	7.725	6.852	124.468	90.883	11.898	10.120	93.594	72.533
2018	11.186	9.318	168.976	99.419	11.873	11.998	167.794	115.153	6.416	5.851	137.238	99.006	7.742	6.820	123.090	90.377	12.057	10.213	91.862	71.510
2019	11.179	9.344	168.833	99.092	11.944	12.072	166.899	114.825	6.393	5.795	136.509	97.992	7.760	6.790	121.752	89.885	12.216	10.306	90.244	70.487
2020	11.174	9.348	168.432	98.541	12.016	12.147	166.107	114.497	6.368	5.822	135.780	98.393	7.778	6.760	120.432	89.406	12.375	10.399	88.533	69.464
2021	11.169	9.376	168.294	98.224	12.088	12.221	165.233	114.169	6.344	5.769	135.050	97.464	7.796	6.732	119.137	88.937	12.534	10.493	86.901	68.441
2022	11.166	9.383	167.897	97.681	12.160	12.296	164.424	113.841	6.320	5.793	134.321	97.786	7.815	6.705	117.861	88.478	12.693	10.586	85.204	67.418
2023	11.164	9.412	167.761	97.368	12.232	12.370	163.564	113.513	6.295	5.743	133.592	96.930	7.833	6.679	116.606	88.027	12.852	10.679	83.560	66.395
2024	11.162	9.419	167.365	96.828	12.304	12.445	162.743	113.184	6.271	5.764	132.863	97.183	7.851	6.654	115.370	87.581	13.011	10.773	81.873	65.372
2025	11.161	9.449	167.231	96.517	12.376	12.519	161.892	112.856	6.247	5.717	132.134	96.393	7.869	6.629	114.152	87.142	13.170	10.866	80.222	64.349
2026	11.161	9.457	166.835	95.978	12.447	12.594	161.065	112.528	6.222	5.735	131.405	96.583	7.887	6.605	112.949	86.707	13.329	10.959	78.541	63.326
2027	11.162	9.488	166.701	95.669	12.519	12.669	160.218	112.200	6.198	5.690	130.675	95.851	7.905	6.582	111.763	86.275	13.488	11.052	76.885	62.303
2028	11.163	9.496	166.305	95.130	12.591	12.743	159.387	111.872	6.173	5.707	129.946	95.987	7.923	6.560	110.591	85.848	13.647	11.146	75.208	61.280
2029	11.165	9.527	166.171	94.821	12.663	12.818	158.544	111.544	6.149	5.664	129.217	95.307	7.941	6.538	109.433	85.422	13.806	11.239	73.548	60.257
2030	11.167	9.535	165.776	94.283	12.735	12.892	157.711	111.216	6.125	5.678	128.488	95.395	7.959	6.517	108.288	85.000	13.964	11.332	71.875	59.234
2031	11.169	9.567	165.642	93.974	12.807	12.967	156.870	110.888	6.100	5.638	127.759	94.759	7.978	6.496	107.155	84.579	14.123	11.426	70.212	58.211
2032	11.172	9.575	165.247	93.436	12.878	13.041	156.034	110.560	6.076	5.650	127.030	94.804	7.996	6.476	106.033	84.160	14.282	11.519	68.541	57.188
2033	11.176	9.607	165.112	93.127	12.950	13.116	155.195	110.232	6.052	5.611	126.300	94.210	8.014	6.456	104.923	83.742	14.441	11.612	66.877	56.165
2034	11.179	9.615	164.717	92.589	13.022	13.190	154.358	109.904	6.027	5.621	125.571	94.217	8.032	6.436	103.822	83.326	14.600	11.706	65.206	55.143
2035	11.183	9.647	164.583	92.280	13.094	13.265	153.519	109.576	6.003	5.585	124.842	93.657	8.050	6.416	102.731	82.910	14.758	11.799	63.541	54.120
2036	11.187	9.655	164.188	91.742	13.166	13.339	152.683	109.248	5.978	5.593	124.113	93.631	8.068	6.397	101.649	82.496	14.917	11.892	61.872	53.097
2037	11.191	9.687	164.054	91.433	13.238	13.414	151.844	108.920	5.954	5.558	123.384	93.103	8.086	6.378	100.575	82.082	15.076	11.985	60.206	52.074
2038	11.196	9.696	163.658	90.895	13.309	13.489	151.007	108.591	5.930	5.564	122.654	93.047	8.104	6.360	99.510	81.668	15.235	12.079	58.537	51.051
2039	11.200	9.727	163.524	90.586	13.381	13.563	150.169	108.263	5.905	5.531	121.925	92.548	8.123	6.341	98.452	81.255	15.393	12.172	56.871	50.028
2040	11.205	9.736	163.129	90.048	13.453	13.638	149.331	107.935	5.881	5.536	121.196	92.465	8.141	6.323	97.400	80.843	15.552	12.265	55.203	49.005

2041	*11.210*	*9.767*	*162.995*	*89.739*	*13.525*	*13.712*	*148.493*	*107.607*	*5.857*	*5.505*	*120.467*	*91.990*	*8.159*	*6.305*	*96.356*	*80.431*	*15.711*	*12.359*	*53.536*	*47.982*
2042	*11.215*	*9.776*	*162.600*	*89.201*	*13.597*	*13.787*	*147.655*	*107.279*	*5.832*	*5.508*	*119.738*	*91.884*	*8.177*	*6.287*	*95.317*	*80.019*	*15.870*	*12.452*	*51.868*	*46.959*
2043	*11.220*	*9.808*	*162.466*	*88.892*	*13.669*	*13.861*	*146.818*	*106.951*	*5.808*	*5.478*	*119.009*	*91.431*	*8.195*	*6.269*	*94.285*	*79.608*	*16.028*	*12.545*	*50.201*	*45.936*
2044	*11.226*	*9.816*	*162.070*	*88.354*	*13.740*	*13.936*	*145.980*	*106.623*	*5.784*	*5.480*	*118.279*	*91.305*	*8.213*	*6.251*	*93.258*	*79.197*	*16.187*	*12.638*	*48.533*	*44.913*
2045	*11.231*	*9.848*	*161.936*	*88.045*	*13.812*	*14.010*	*145.142*	*106.295*	*5.759*	*5.451*	*117.550*	*90.871*	*8.231*	*6.234*	*92.236*	*78.786*	*16.346*	*12.732*	*46.866*	*43.890*
2046	*11.236*	*9.857*	*161.541*	*87.507*	*13.884*	*14.085*	*144.304*	*105.967*	*5.735*	*5.452*	*116.821*	*90.727*	*8.249*	*6.216*	*91.218*	*78.375*	*16.504*	*12.825*	*45.198*	*42.867*
2047	*11.242*	*9.888*	*161.407*	*87.199*	*13.956*	*14.160*	*143.466*	*105.639*	*5.710*	*5.424*	*116.092*	*90.310*	*8.268*	*6.199*	*90.206*	*77.964*	*16.663*	*12.918*	*43.531*	*41.844*
2048	*11.247*	*9.897*	*161.012*	*86.661*	*14.028*	*14.234*	*142.629*	*105.311*	*5.686*	*5.424*	*115.363*	*90.150*	*8.286*	*6.182*	*89.198*	*77.554*	*16.821*	*13.012*	*41.864*	*40.821*
2049	*11.253*	*9.929*	*160.878*	*86.352*	*14.100*	*14.309*	*141.791*	*104.983*	*5.662*	*5.397*	*114.634*	*89.748*	*8.304*	*6.165*	*88.194*	*77.143*	*16.980*	*13.105*	*40.196*	*39.798*
2050	*11.259*	*9.937*	*160.482*	*85.814*	*14.171*	*14.383*	*140.953*	*104.655*	*5.637*	*5.396*	*113.904*	*89.573*	*8.322*	*6.148*	*87.193*	*76.733*	*17.139*	*13.198*	*38.529*	*38.776*
2051	*11.265*	*9.969*	*160.348*	*85.505*	*14.243*	*14.458*	*140.115*	*104.327*	*5.613*	*5.370*	*113.175*	*89.185*	*8.340*	*6.131*	*86.196*	*76.322*	*17.297*	*13.291*	*36.861*	*37.753*
2052	*11.270*	*9.978*	*159.953*	*84.967*	*14.315*	*14.532*	*139.278*	*103.998*	*5.589*	*5.368*	*112.446*	*88.998*	*8.358*	*6.114*	*85.203*	*75.912*	*17.456*	*13.385*	*35.194*	*36.730*
2053	*11.276*	*10.009*	*159.819*	*84.658*	*14.387*	*14.607*	*138.440*	*103.670*	*5.564*	*5.343*	*111.717*	*88.622*	*8.376*	*6.097*	*84.213*	*75.502*	*17.614*	*13.478*	*33.527*	*35.707*
2054	*11.282*	*10.018*	*159.424*	*84.120*	*14.459*	*14.681*	*137.602*	*103.342*	*5.540*	*5.340*	*110.988*	*88.423*	*8.394*	*6.080*	*83.225*	*75.091*	*17.773*	*13.571*	*31.859*	*34.684*
2055	*11.288*	*10.050*	*159.290*	*83.811*	*14.531*	*14.756*	*136.764*	*103.014*	*5.515*	*5.316*	*110.259*	*88.057*	*8.412*	*6.063*	*82.241*	*74.681*	*17.931*	*13.665*	*30.192*	*33.661*
2056	*11.294*	*10.058*	*158.894*	*83.273*	*14.602*	*14.830*	*135.926*	*102.686*	*5.491*	*5.312*	*109.529*	*87.849*	*8.431*	*6.046*	*81.259*	*74.271*	*18.090*	*13.758*	*28.524*	*32.638*
2057	*11.300*	*10.090*	*158.760*	*82.964*	*14.674*	*14.905*	*135.089*	*102.358*	*5.467*	*5.289*	*108.800*	*87.492*	*8.449*	*6.029*	*80.279*	*73.861*	*18.248*	*13.851*	*26.857*	*31.615*
2058	*11.306*	*10.099*	*158.365*	*82.426*	*14.746*	*14.980*	*134.251*	*102.030*	*5.442*	*5.284*	*108.071*	*87.275*	*8.467*	*6.013*	*79.302*	*73.451*	*18.407*	*13.944*	*25.189*	*30.592*
2059	*11.312*	*10.130*	*158.231*	*82.117*	*14.818*	*15.054*	*133.413*	*101.702*	*5.418*	*5.262*	*107.342*	*86.927*	*8.485*	*5.996*	*78.327*	*73.040*	*18.565*	*14.038*	*23.522*	*29.569*
2060	*11.318*	*10.139*	*157.836*	*81.580*	*14.890*	*15.129*	*132.575*	*101.374*	*5.394*	*5.256*	*106.613*	*86.702*	*8.503*	*5.979*	*77.355*	*72.630*	*18.724*	*14.131*	*21.854*	*28.546*
2061	*11.324*	*10.171*	*157.701*	*81.271*	*14.962*	*15.203*	*131.737*	*101.046*	*5.369*	*5.234*	*105.884*	*86.361*	*8.521*	*5.963*	*76.384*	*72.220*	*18.882*	*14.224*	*20.187*	*27.523*
2062	*11.330*	*10.179*	*157.306*	*80.733*	*15.033*	*15.278*	*130.900*	*100.718*	*5.345*	*5.228*	*105.154*	*86.129*	*8.539*	*5.946*	*75.414*	*71.810*	*19.041*	*14.318*	*18.520*	*26.500*
2063	*11.336*	*10.211*	*157.172*	*80.424*	*15.105*	*15.352*	*130.062*	*100.390*	*5.321*	*5.207*	*104.425*	*85.795*	*8.557*	*5.929*	*74.447*	*71.400*	*19.199*	*14.411*	*16.852*	*25.477*
2064	*11.342*	*10.220*	*156.777*	*79.886*	*15.177*	*15.427*	*129.224*	*100.062*	*5.296*	*5.201*	*103.696*	*85.557*	*8.576*	*5.913*	*73.481*	*70.990*	*19.358*	*14.504*	*15.185*	*24.454*
2065	*11.348*	*10.251*	*156.643*	*79.577*	*15.249*	*15.501*	*128.386*	*99.733*	*5.272*	*5.180*	*102.967*	*85.228*	*8.594*	*5.896*	*72.517*	*70.580*	*19.516*	*14.598*	*13.517*	*23.431*
2066	*11.354*	*10.260*	*156.248*	*79.039*	*15.321*	*15.576*	*127.548*	*99.405*	*5.247*	*5.173*	*102.238*	*84.985*	*8.612*	*5.880*	*71.554*	*70.170*	*19.674*	*14.691*	*11.850*	*22.409*
2067	*11.360*	*10.292*	*156.113*	*78.730*	*15.393*	*15.651*	*126.711*	*99.077*	*5.223*	*5.153*	*101.508*	*84.661*	*8.630*	*5.863*	*70.593*	*69.760*	*19.833*	*14.784*	*10.182*	*21.386*
2068	*11.366*	*10.301*	*155.718*	*78.192*	*15.464*	*15.725*	*125.873*	*98.749*	*5.199*	*5.145*	*100.779*	*84.413*	*8.648*	*5.847*	*69.632*	*69.350*	*19.991*	*14.877*	*8.515*	*20.363*
2069	*11.372*	*10.332*	*155.584*	*77.883*	*15.536*	*15.800*	*125.035*	*98.421*	*5.174*	*5.126*	*100.050*	*84.094*	*8.666*	*5.830*	*68.673*	*68.940*	*20.150*	*14.971*	*6.848*	*19.340*
2070	*11.379*	*10.341*	*155.189*	*77.345*	*15.608*	*15.874*	*124.197*	*98.093*	*5.150*	*5.117*	*99.321*	*83.842*	*8.684*	*5.814*	*67.715*	*68.530*	*20.308*	*15.064*	*5.180*	*18.317*
2071	*11.385*	*10.372*	*155.055*	*77.036*	*15.680*	*15.949*	*123.360*	*97.765*	*5.126*	*5.098*	*98.592*	*83.527*	*8.702*	*5.797*	*66.758*	*68.120*	*20.466*	*15.157*	*3.513*	*17.294*
2072	*11.391*	*10.381*	*154.660*	*76.498*	*15.752*	*16.023*	*122.522*	*97.437*	*5.101*	*5.090*	*97.863*	*83.271*	*8.721*	*5.781*	*65.803*	*67.710*	*20.624*	*15.251*	*1.845*	*16.271*
2073	*11.397*	*10.413*	*154.525*	*76.189*	*15.824*	*16.098*	*121.684*	*97.109*	*5.077*	*5.071*	*97.133*	*82.959*	*8.739*	*5.764*	*64.848*	*67.300*	*20.783*	*15.344*	*0.178*	*15.248*

2074	11.403	10.422	154.130	75.652	15.895	16.172	120.846	96.781	5.052	5.062	96.404	82.700	8.757	5.748	63.893	66.890	20.941	15.437	-1.490	14.225
2075	11.409	10.453	153.996	75.343	15.967	16.247	120.008	96.453	5.028	5.044	95.675	82.391	8.775	5.731	62.940	66.480	21.099	15.530	-3.157	13.202
2076	11.415	10.462	153.601	74.805	16.039	16.322	119.171	96.125	5.004	5.034	94.946	82.129	8.793	5.715	61.988	66.070	21.258	15.624	-4.824	12.179
2077	11.422	10.493	153.467	74.496	16.111	16.396	118.333	95.797	4.979	5.016	94.217	81.823	8.811	5.698	61.036	65.660	21.416	15.717	-6.492	11.156
2078	11.428	10.502	153.072	73.958	16.183	16.471	117.495	95.469	4.955	5.007	93.488	81.558	8.829	5.682	60.085	65.250	21.574	15.810	-8.159	10.133
2079	11.434	10.534	152.937	73.649	16.255	16.545	116.657	95.140	4.931	4.989	92.758	81.255	8.847	5.665	59.134	64.839	21.732	15.904	-9.827	9.110
2080	11.440	10.543	152.542	73.111	16.326	16.620	115.819	94.812	4.906	4.979	92.029	80.988	8.865	5.649	58.184	64.429	21.891	15.997	-11.494	8.087
2081	11.446	10.574	152.408	72.802	16.398	16.694	114.982	94.484	4.882	4.962	91.300	80.686	8.884	5.632	57.235	64.019	22.049	16.090	-13.162	7.064
2082	11.452	10.583	152.013	72.264	16.470	16.769	114.144	94.156	4.857	4.951	90.571	80.417	8.902	5.616	56.286	63.609	22.207	16.183	-14.829	6.042
2083	11.459	10.614	151.879	71.955	16.542	16.843	113.306	93.828	4.833	4.934	89.842	80.118	8.920	5.600	55.338	63.199	22.365	16.277	-16.496	5.019
2084	11.465	10.623	151.483	71.417	16.614	16.918	112.468	93.500	4.809	4.924	89.113	79.847	8.938	5.583	54.390	62.789	22.523	16.370	-18.164	3.996
2085	11.471	10.655	151.349	71.108	16.686	16.992	111.630	93.172	4.784	4.907	88.383	79.549	8.956	5.567	53.443	62.379	22.681	16.463	-19.831	2.973
2086	11.477	10.664	150.954	70.571	16.757	17.067	110.793	92.844	4.760	4.896	87.654	79.277	8.974	5.550	52.496	61.969	22.840	16.557	-21.499	1.950
2087	11.483	10.695	150.820	70.262	16.829	17.142	109.955	92.516	4.736	4.880	86.925	78.981	8.992	5.534	51.549	61.559	22.998	16.650	-23.166	0.927
2088	11.489	10.704	150.425	69.724	16.901	17.216	109.117	92.188	4.711	4.869	86.196	78.707	9.010	5.517	50.603	61.149	23.156	16.743	-24.834	-0.096
2089	11.496	10.735	150.291	69.415	16.973	17.291	108.279	91.860	4.687	4.852	85.467	78.412	9.029	5.501	49.657	60.739	23.314	16.837	-26.501	-1.119
2090	11.502	10.744	149.895	68.877	17.045	17.365	107.442	91.532	4.663	4.841	84.738	78.137	9.047	5.485	48.712	60.329	23.472	16.930	-28.168	-2.142
2091	11.508	10.776	149.761	68.568	17.117	17.440	106.604	91.204	4.638	4.825	84.008	77.843	9.065	5.468	47.766	59.919	23.630	17.023	-29.836	-3.165
2092	11.514	10.785	149.366	68.030	17.188	17.514	105.766	90.876	4.614	4.813	83.279	77.567	9.083	5.452	46.821	59.509	23.788	17.116	-31.503	-4.188
2093	11.520	10.816	149.232	67.721	17.260	17.589	104.928	90.547	4.589	4.797	82.550	77.274	9.101	5.435	45.877	59.099	23.946	17.210	-33.171	-5.211
2094	11.526	10.825	148.837	67.183	17.332	17.663	104.090	90.219	4.565	4.786	81.821	76.997	9.119	5.419	44.932	58.689	24.104	17.303	-34.838	-6.234
2095	11.533	10.856	148.702	66.874	17.404	17.738	103.253	89.891	4.541	4.770	81.092	76.705	9.137	5.402	43.988	58.279	24.262	17.396	-36.506	-7.257
2096	11.539	10.865	148.307	66.336	17.476	17.813	102.415	89.563	4.516	4.758	80.362	76.427	9.155	5.386	43.044	57.869	24.420	17.490	-38.173	-8.280
2097	11.545	10.897	148.173	66.027	17.548	17.887	101.577	89.235	4.492	4.743	79.633	76.136	9.174	5.370	42.100	57.459	24.578	17.583	-39.840	-9.303
2098	11.551	10.906	147.778	65.489	17.620	17.962	100.739	88.907	4.468	4.731	78.904	75.857	9.192	5.353	41.157	57.049	24.736	17.676	-41.508	-10.325
2099	11.557	10.937	147.644	65.180	17.691	18.036	99.901	88.579	4.443	4.715	78.175	75.567	9.210	5.337	40.214	56.639	24.894	17.769	-43.175	-11.348
2100	11.564	10.946	147.249	64.643	17.763	18.111	99.064	88.251	4.419	4.703	77.446	75.288	9.228	5.320	39.270	56.229	25.052	17.863	-44.843	-12.371
2101	11.570	10.977	147.114	64.334	17.835	18.185	98.226	87.923	4.394	4.688	76.717	74.998	9.246	5.304	38.327	55.819	25.210	17.956	-46.510	-13.394
2102	11.576	10.986	146.719	63.796	17.907	18.260	97.388	87.595	4.370	4.676	75.987	74.718	9.264	5.288	37.385	55.409	25.368	18.049	-48.178	-14.417
2103	11.582	11.018	146.585	63.487	17.979	18.334	96.550	87.267	4.346	4.660	75.258	74.429	9.282	5.271	36.442	54.999	25.526	18.143	-49.845	-15.440
2104	11.588	11.027	146.190	62.949	18.051	18.409	95.712	86.939	4.321	4.648	74.529	74.148	9.300	5.255	35.499	54.589	25.684	18.236	-51.512	-16.463
2105	11.594	11.058	146.056	62.640	18.122	18.483	94.875	86.611	4.297	4.633	73.800	73.860	9.318	5.238	34.557	54.179	25.842	18.329	-53.180	-17.486
2106	11.601	11.067	145.661	62.102	18.194	18.558	94.037	86.283	4.273	4.620	73.071	73.579	9.337	5.222	33.615	53.769	25.999	18.422	-54.847	-18.509

2107	*11.607*	*11.098*	*145.526*	*61.793*	*18.266*	*18.633*	*93.199*	*85.955*	*4.248*	*4.605*	*72.342*	*73.291*	*9.355*	*5.206*	*32.673*	*53.359*	*26.157*	*18.516*	*-56.515*	*-19.532*
2108	*11.613*	*11.108*	*145.131*	*61.255*	*18.338*	*18.707*	*92.361*	*85.626*	*4.224*	*4.593*	*71.612*	*73.009*	*9.373*	*5.189*	*31.730*	*52.949*	*26.315*	*18.609*	*-58.182*	*-20.555*
2109	*11.619*	*11.139*	*144.997*	*60.946*	*18.410*	*18.782*	*91.524*	*85.298*	*4.200*	*4.578*	*70.883*	*72.721*	*9.391*	*5.173*	*30.788*	*52.539*	*26.473*	*18.702*	*-59.850*	*-21.578*
2110	*11.625*	*11.148*	*144.602*	*60.408*	*18.482*	*18.856*	*90.686*	*84.970*	*4.175*	*4.565*	*70.154*	*72.439*	*9.409*	*5.156*	*29.847*	*52.129*	*26.631*	*18.796*	*-61.517*	*-22.601*

(Abbreviations: CRC, colorectal cancer; SDI, sociodemographic index)
Note: The numbers in normal font are the data downloaded from GBD and GLOBOCAN. The italicized numbers indicate the forecasted incidence rates.

Supplemental Table 3. The average annual rates of change in death rates

Region	Group	Gender	Average AROC of available data	Average AROC of available data and 30-year forecast	Average AROC of available data and 50-year forecast	Average AROC of available data and 90-year forecast
High SDI	YOCRC	Male	-0·18%	-0·42%	-0·51%	-0·67%
		Female	-0·32%	-0·41%	-0·45%	-0·52%
	LOCRC	Male	-1·23%	-1·22%	-1·32%	-2·06%
		Female	-1·86%	-1·82%	-2·10%	-4·03%
Australia	YOCRC	Male	-0·33%	-0·97%	-1·80%	-7·46%
		Female	-1·30%	-2·56%	-1·96%	-0·49%
	LOCRC	Male	-1·39%	1·26%	1·77%	1·80%
		Female	-2·33%	-0·25%	0·46%	0·81%
Singapore	YOCRC	Male	4·89%	3·10%	2·50%	1·80%
		Female	6·73%	4·34%	3·53%	2·59%
	LOCRC	Male	2·45%	1·01%	0·36%	-2·25%
		Female	2·33%	0·09%	0·35%	1·70%
Switzerland	YOCRC	Male	1·68%	0·96%	0·67%	0·28%
		Female	0·62%	0·13%	-0·09%	-0·52%
	LOCRC	Male	-0·84%	-1·17%	-1·58%	-0·44%
		Female	-1·13%	-1·42%	-1·89%	-1·81%
USA	YOCRC	Male	-0·60%	-0·49%	-0·44%	-0·40%
		Female	-1·35%	-1·31%	-1·38%	-2·08%
	LOCRC	Male	-1·87%	-3·23%	-1·86%	-0·88%
		Female	-2·68%	-3·22%	-2·01%	-1·03%

(Abbreviations: AROC, Annual rate of change; LOCRC, Late-onset colorectal cancer; SDI, Socio-demographic index; YOCRC, Young-onset colorectal cancer)

Supplemental Table 4. Death rate data and forecasts

Year	High SDI				Australia				Singapore				Switzerland				United States			
	Young Onset CRC		Late Onset CRC		Young Onset CRC		Late Onset CRC		Young Onset CRC		Late Onset CRC		Young Onset CRC		Late Onset CRC		Young Onset CRC		Late Onset CRC	
	Male	Female	Male	Female	Male	Female	Male	Female	Male	Female	Male	Female	Male	Female	Male	Female	Male	Female	Male	Female
1955					3.345	4.355	62.101	62.064					2.768	3.206	67.712	47.127				
1956					3.460	4.414	62.128	60.086					3.247	2.393	66.238	48.372				
1957					3.573	4.695	68.294	58.023					1.817	2.935	61.483	48.349				
1958					4.134	4.347	62.974	62.784					1.962	2.205	64.237	43.877	3.435	4.030	65.279	57.643
1959					3.412	4.437	64.941	57.609					1.857	1.872	68.280	47.625	3.397	4.240	65.114	57.230
1960					3.828	4.899	61.430	56.069					2.224	2.078	61.779	42.092	3.321	3.929	65.117	57.200
1961					3.295	3.981	57.547	58.927					2.340	1.747	64.720	41.210	3.322	4.106	65.444	56.948
1962					4.276	4.189	59.687	60.500					2.134	1.941	62.601	44.597	3.274	3.709	64.745	57.390
1963					4.229	4.938	61.121	56.603					2.080	1.975	64.929	42.992	3.123	3.661	64.422	56.016
1964					3.840	4.321	60.545	52.323					2.604	1.647	65.491	37.983	3.205	3.557	64.261	56.058
1965					3.568	4.710	59.865	52.654					1.763	1.192	68.526	43.206	3.082	3.403	64.926	54.977
1966					3.989	3.688	59.694	56.827	2.204	1.681	28.677	33.266	1.939	1.657	64.900	47.703	3.083	3.405	64.397	55.900
1967					4.105	4.075	57.420	58.612	3.425	2.085	44.894	26.949	1.770	1.633	70.419	45.909	2.879	3.304	64.218	54.547
1968					3.718	4.195	62.417	54.831	2.487	1.341	37.447	37.453	1.943	1.880	68.880	46.057	2.981	3.331	64.641	54.564
1969					3.870	4.179	58.218	56.900	1.607	2.367	27.318	18.083	2.379	1.460	79.039	46.281	2.814	3.069	65.074	53.626
1970					3.835	4.697	62.089	56.825	2.302	2.026	41.906	32.702	2.480	1.314	69.476	47.812	2.735	2.863	64.309	54.654
1971					3.605	4.052	62.339	55.475	1.854	1.757	44.551	27.523	2.138	2.477	70.684	46.703	2.685	2.958	64.073	52.866
1972					3.633	3.821	67.131	56.711	3.038	2.439	48.837	51.136	1.674	1.359	76.980	41.977	2.634	2.848	63.905	52.837
1973					3.932	3.789	63.355	56.108	3.453	2.713	40.030	37.154	2.113	1.287	67.965	47.504	2.500	2.665	62.833	53.338
1974					4.049	3.147	70.817	56.953	3.003	1.572	50.847	51.380	1.466	1.536	69.493	44.487	2.604	2.619	65.448	53.368
1975					4.015	4.115	71.310	57.527	2.421	1.686	55.954	41.854	1.842	2.038	67.612	41.502	2.529	2.422	64.016	52.259
1976					3.764	3.461	66.464	55.945	3.288	2.285	57.183	54.745	1.901	1.908	70.178	48.115	2.359	2.422	64.961	53.121
1977					3.958	3.400	72.049	55.811	2.588	1.901	61.779	38.979	2.216	1.590	66.326	41.487	2.371	2.294	66.126	51.917
1978					4.085	3.364	74.783	55.036	2.971	1.853	62.582	50.754	2.528	1.590	59.901	46.455	2.342	2.167	66.344	52.557
1979					3.289	3.132	74.791	54.277	2.030	3.759	61.185	49.867	2.268	1.715	64.689	45.176	2.187	2.077	65.419	50.627
1980					3.446	3.843	71.636	57.340	2.060	1.439	58.366	58.978	1.567	1.203	67.912	43.206	2.186	1.967	65.766	50.321
1981					3.429	3.627	74.833	53.175	1.345	3.239	56.151	62.420	2.173	1.696	65.948	40.870	2.140	1.899	65.528	48.808

1982					3.467	2.978	76.524	55.954	2.511	2.763	59.729	56.382	1.964	1.805	67.326	40.154	2.077	1.879	65.772	49.241
1983					2.603	3.492	82.538	57.225	2.341	3.128	70.270	45.756	2.001	1.663	66.516	43.642	2.115	1.836	66.739	49.190
1984					3.043	3.068	81.037	57.919	2.951	3.486	52.848	41.545	2.338	1.708	62.387	41.969	2.065	1.808	68.066	50.416
1985					3.557	2.904	80.868	59.596	3.172	1.857	63.927	64.023	2.193	1.390	63.693	40.904	2.137	1.895	67.746	48.702
1986					3.605	3.183	82.270	59.621	2.254	3.665	62.813	50.055	1.469	1.620	54.936	38.986	2.054	1.836	66.751	48.135
1987					3.182	2.990	83.739	59.278	2.839	2.970	60.870	46.699	1.688	1.429	62.699	36.552	2.215	1.901	66.540	47.458
1988					2.684	2.503	84.187	57.554	1.509	2.212	56.277	43.915	2.654	1.478	64.342	40.674	2.250	1.856	64.839	46.423
1989					2.942	2.609	81.069	54.926	3.219	2.823	85.550	45.178	1.488	1.468	61.101	38.035	2.045	1.729	64.497	44.680
1990	2.915	2.446	70.501	48.028	2.830	2.385	78.765	56.377	3.109	3.475	60.379	46.670	1.818	2.101	56.727	37.636	2.147	1.898	63.439	43.761
1991	2.933	2.456	70.836	47.857	2.879	2.612	78.316	52.957	2.475	2.681	70.775	43.478	2.030	1.620	54.811	35.496	2.166	1.919	61.891	43.199
1992	2.961	2.463	71.460	47.660	2.468	2.732	79.072	52.517	2.783	2.147	53.546	60.164	2.185	1.320	56.785	34.307	2.274	1.907	61.152	42.029
1993	3.001	2.497	72.429	47.933	2.914	2.427	77.415	53.400	2.858	2.339	72.084	52.069	1.839	1.539	63.297	39.223	2.242	1.916	60.769	42.465
1994	3.016	2.498	72.411	47.383	2.937	2.279	85.677	55.101	3.385	2.872	68.981	48.686	1.831	2.040	57.555	35.051	2.375	2.037	60.005	41.184
1995	3.053	2.533	72.550	46.925	2.648	2.369	76.546	50.053	3.441	3.151	75.645	45.186	2.213	1.749	52.666	31.729	2.368	2.003	58.425	40.378
1996	3.061	2.540	71.528	45.821	2.366	2.544	80.175	49.755	3.497	2.985	63.613	54.330	1.887	1.468	49.319	29.895	2.370	2.031	56.209	37.998
1997	3.053	2.529	69.605	44.475	2.751	2.134	76.588	50.870	2.277	2.626	66.853	43.998	1.504	1.641	49.667	30.302	2.389	2.121	54.147	36.462
1998	3.043	2.508	68.691	43.542	2.928	2.730	70.772	47.774	3.010	3.889	62.974	50.475	1.845	1.531	48.828	27.733	2.313	2.029	52.746	36.240
1999	3.036	2.505	67.745	42.906	1.810	2.472	69.819	42.109	1.497	2.036	62.927	46.069	1.121	1.534	49.491	27.007	2.405	2.187	50.256	35.027
2000	3.075	2.505	66.217	42.015	2.648	2.215	67.271	41.619	1.918	3.192	51.536	44.335	2.021	1.421	48.973	29.714	2.509	2.118	49.220	33.704
2001	3.105	2.513	64.797	41.015	2.406	2.367	64.472	40.922	2.970	2.216	67.383	38.209	1.287	1.415	46.981	29.139	2.632	2.225	47.709	32.836
2002	3.118	2.494	63.823	40.077	2.106	2.351	57.299	39.693	2.845	1.776	62.073	45.075	1.616	1.858	44.137	28.444	2.650	2.183	47.188	31.437
2003	3.126	2.484	62.843	39.140	2.249	2.456	55.881	34.107	3.134	2.787	57.749	35.267	1.440	1.734	43.482	27.222	2.523	2.090	44.219	30.140
2004	3.103	2.456	61.102	37.688	2.148	2.113	49.377	31.124	2.905	2.342	56.456	38.866	2.090	1.277	41.369	25.784	2.421	2.078	40.963	27.776
2005	3.103	2.446	60.099	36.711	2.124	1.893	47.057	28.239	2.155	2.321	54.550	34.527	1.964	1.432	45.896	23.546	2.423	2.086	39.586	26.537
2006	3.086	2.419	58.634	35.614	2.144	1.726	45.128	25.762	3.144	2.884	49.002	34.280	1.650	1.480	41.353	23.625	2.385	1.916	37.659	24.176
2007	3.064	2.400	57.899	34.902	2.007	1.709	44.387	25.332	2.156	2.223	50.025	29.748	1.644	1.445	40.756	23.329	2.378	1.899	36.184	23.065
2008	3.063	2.411	57.442	34.340	1.975	1.674	42.328	24.292	2.606	2.598	52.997	33.599	1.643	1.427	40.290	22.854	2.371	1.879	34.714	21.949
2009	3.053	2.413	56.849	33.671	1.945	1.623	40.251	23.128	2.584	2.611	52.298	32.429	1.635	1.425	39.789	22.738	2.364	1.861	33.256	20.841
2010	3.007	2.376	56.080	33.001	1.918	1.606	38.250	22.005	2.601	2.573	51.789	32.226	1.626	1.415	39.279	22.306	2.358	1.841	31.810	19.733
2011	2.963	2.361	55.436	32.661	1.889	1.559	36.277	20.887	2.601	2.607	51.198	31.406	1.618	1.402	38.773	22.051	2.351	1.823	30.375	18.630
2012	2.905	2.324	54.703	32.135	1.860	1.527	34.349	19.761	2.603	2.608	50.641	30.929	1.610	1.392	38.267	21.754	2.345	1.804	28.951	17.530
2013	2.868	2.301	54.116	31.789	1.832	1.495	32.455	18.637	2.603	2.607	50.070	30.235	1.602	1.383	37.761	21.416	2.338	1.786	27.537	16.433
2014	2.852	2.281	52.180	30.188	1.803	1.455	30.597	17.514	2.604	2.619	49.505	29.663	1.594	1.372	37.255	21.146	2.332	1.767	26.134	15.339

2015	2.829	2.269	51.548	29.719	1.775	1.424	28.770	16.390	2.604	2.623	48.937	29.015	1.586	1.362	36.749	20.820	2.326	1.749	24.741	14.249
2016	2.807	2.255	50.932	29.255	1.746	1.387	26.973	15.266	2.605	2.628	48.371	28.410	1.578	1.351	36.242	20.523	2.320	1.730	23.359	13.161
2017	2.787	2.246	50.332	28.799	1.717	1.351	25.204	14.142	2.605	2.635	47.804	27.778	1.569	1.341	35.736	20.220	2.313	1.712	21.986	12.077
2018	2.768	2.233	49.745	28.350	1.689	1.318	23.462	13.018	2.605	2.640	47.237	27.161	1.561	1.331	35.230	19.910	2.307	1.694	20.623	10.996
2019	2.749	2.224	49.170	27.908	1.660	1.281	21.744	11.894	2.605	2.645	46.670	26.535	1.553	1.320	34.724	19.611	2.301	1.676	19.269	9.918
2020	2.731	2.212	48.607	27.471	1.632	1.247	20.049	10.770	2.606	2.651	46.103	25.915	1.545	1.310	34.218	19.303	2.295	1.658	17.925	8.843
2021	2.714	2.203	48.052	27.040	1.603	1.211	18.376	9.647	2.606	2.657	45.536	25.291	1.537	1.300	33.712	19.000	2.289	1.640	16.590	7.770
2022	2.696	2.191	47.507	26.614	1.575	1.176	16.722	8.523	2.606	2.663	44.970	24.669	1.529	1.289	33.206	18.696	2.284	1.622	15.264	6.701
2023	2.679	2.183	46.969	26.192	1.546	1.141	15.087	7.399	2.606	2.668	44.403	24.046	1.521	1.279	32.700	18.390	2.278	1.604	13.946	5.635
2024	2.663	2.171	46.437	25.775	1.517	1.106	13.470	6.275	2.607	2.674	43.836	23.423	1.513	1.269	32.194	18.087	2.272	1.586	12.638	4.572
2025	2.646	2.162	45.912	25.362	1.489	1.071	11.869	5.151	2.607	2.680	43.269	22.800	1.505	1.259	31.687	17.781	2.266	1.568	11.338	3.512
2026	2.630	2.150	45.392	24.953	1.460	1.036	10.284	4.027	2.607	2.685	42.702	22.178	1.497	1.248	31.181	17.477	2.260	1.550	10.046	2.455
2027	2.613	2.142	44.877	24.547	1.432	1.000	8.713	2.904	2.607	2.691	42.135	21.555	1.489	1.238	30.675	17.173	2.255	1.532	8.762	1.401
2028	2.597	2.130	44.366	24.145	1.403	0.965	7.155	1.780	2.608	2.697	41.568	20.932	1.481	1.228	30.169	16.868	2.249	1.515	7.486	0.349
2029	2.581	2.122	43.859	23.745	1.374	0.930	5.610	0.656	2.608	2.702	41.001	20.309	1.473	1.217	29.663	16.564	2.244	1.497	6.218	-0.700
2030	2.565	2.110	43.355	23.348	1.346	0.895	4.076	-0.468	2.608	2.708	40.435	19.687	1.464	1.207	29.157	16.259	2.238	1.479	4.958	-1.745
2031	2.548	2.102	42.855	22.954	1.317	0.860	2.554	-1.592	2.608	2.714	39.868	19.064	1.456	1.197	28.651	15.955	2.232	1.462	3.705	-2.788
2032	2.532	2.090	42.357	22.562	1.289	0.825	1.042	-2.716	2.609	2.720	39.301	18.441	1.448	1.186	28.145	15.650	2.227	1.444	2.460	-3.829
2033	2.516	2.081	41.861	22.172	1.260	0.790	-0.461	-3.839	2.609	2.725	38.734	17.818	1.440	1.176	27.639	15.346	2.221	1.427	1.221	-4.866
2034	2.500	2.070	41.368	21.784	1.232	0.755	-1.955	-4.963	2.609	2.731	38.167	17.196	1.432	1.166	27.132	15.041	2.216	1.409	-0.010	-5.901
2035	2.484	2.061	40.876	21.398	1.203	0.719	-3.440	-6.087	2.609	2.737	37.600	16.573	1.424	1.155	26.626	14.737	2.210	1.392	-1.234	-6.933
2036	2.468	2.049	40.386	21.014	1.174	0.684	-4.918	-7.211	2.610	2.742	37.033	15.950	1.416	1.145	26.120	14.432	2.205	1.374	-2.451	-7.962
2037	2.452	2.041	39.897	20.632	1.146	0.649	-6.388	-8.335	2.610	2.748	36.467	15.327	1.408	1.135	25.614	14.128	2.200	1.357	-3.661	-8.989
2038	2.436	2.029	39.410	20.251	1.117	0.614	-7.851	-9.459	2.610	2.754	35.900	14.705	1.400	1.124	25.108	13.823	2.194	1.339	-4.865	-10.013
2039	2.420	2.021	38.924	19.871	1.089	0.579	-9.308	-10.582	2.610	2.759	35.333	14.082	1.392	1.114	24.602	13.519	2.189	1.322	-6.063	-11.034
2040	2.404	2.009	38.439	19.493	1.060	0.544	-10.759	-11.706	2.611	2.765	34.766	13.459	1.384	1.104	24.096	13.214	2.183	1.305	-7.254	-12.053
2041	2.387	2.000	37.956	19.116	1.032	0.509	-12.205	-12.830	2.611	2.771	34.199	12.836	1.376	1.093	23.590	12.910	2.178	1.287	-8.439	-13.069
2042	2.371	1.989	37.472	18.740	1.003	0.473	-13.645	-13.954	2.611	2.776	33.632	12.214	1.368	1.083	23.084	12.605	2.173	1.270	-9.618	-14.082
2043	2.355	1.980	36.990	18.365	0.974	0.438	-15.080	-15.078	2.611	2.782	33.065	11.591	1.359	1.073	22.577	12.301	2.167	1.253	-10.791	-15.093
2044	2.339	1.969	36.508	17.991	0.946	0.403	-16.511	-16.202	2.612	2.788	32.498	10.968	1.351	1.062	22.071	11.996	2.162	1.236	-11.958	-16.102
2045	2.323	1.960	36.027	17.618	0.917	0.368	-17.937	-17.325	2.612	2.793	31.932	10.345	1.343	1.052	21.565	11.692	2.157	1.218	-13.119	-17.108
2046	2.307	1.948	35.547	17.245	0.889	0.333	-19.359	-18.449	2.612	2.799	31.365	9.723	1.335	1.042	21.059	11.387	2.151	1.201	-14.275	-18.111
2047	2.291	1.940	35.066	16.874	0.860	0.298	-20.778	-19.573	2.612	2.805	30.798	9.100	1.327	1.031	20.553	11.083	2.146	1.184	-15.425	-19.112

2048	*2.275*	*1.928*	*34.587*	*16.503*	*0.831*	*0.263*	*-22.193*	*-20.697*	*2.613*	*2.811*	*30.231*	*8.477*	*1.319*	*1.021*	*20.047*	*10.778*	*2.141*	*1.167*	*-16.570*	*-20.110*
2049	*2.259*	*1.920*	*34.107*	*16.133*	*0.803*	*0.228*	*-23.605*	*-21.821*	*2.613*	*2.816*	*29.664*	*7.854*	*1.311*	*1.011*	*19.541*	*10.474*	*2.136*	*1.150*	*-17.710*	*-21.106*
2050	*2.243*	*1.908*	*33.628*	*15.763*	*0.774*	*0.192*	*-25.014*	*-22.945*	*2.613*	*2.822*	*29.097*	*7.232*	*1.303*	*1.001*	*19.035*	*10.169*	*2.130*	*1.133*	*-18.844*	*-22.100*
2051	*2.227*	*1.899*	*33.150*	*15.394*	*0.746*	*0.157*	*-26.420*	*-24.068*	*2.613*	*2.828*	*28.530*	*6.609*	*1.295*	*0.990*	*18.528*	*9.865*	*2.125*	*1.116*	*-19.973*	*-23.091*
2052	*2.211*	*1.888*	*32.671*	*15.025*	*0.717*	*0.122*	*-27.823*	*-25.192*	*2.613*	*2.833*	*27.964*	*5.986*	*1.287*	*0.980*	*18.022*	*9.560*	*2.120*	*1.099*	*-21.097*	*-24.080*
2053	*2.195*	*1.879*	*32.193*	*14.657*	*0.689*	*0.087*	*-29.224*	*-26.316*	*2.614*	*2.839*	*27.397*	*5.363*	*1.279*	*0.970*	*17.516*	*9.256*	*2.115*	*1.082*	*-22.217*	*-25.066*
2054	*2.179*	*1.867*	*31.715*	*14.290*	*0.660*	*0.052*	*-30.622*	*-27.440*	*2.614*	*2.845*	*26.830*	*4.741*	*1.271*	*0.959*	*17.010*	*8.951*	*2.109*	*1.064*	*-23.331*	*-26.050*
2055	*2.163*	*1.859*	*31.237*	*13.922*	*0.631*	*0.017*	*-32.019*	*-28.564*	*2.614*	*2.850*	*26.263*	*4.118*	*1.263*	*0.949*	*16.504*	*8.647*	*2.104*	*1.047*	*-24.441*	*-27.032*
2056	*2.147*	*1.847*	*30.760*	*13.556*	*0.603*	*-0.018*	*-33.413*	*-29.688*	*2.614*	*2.856*	*25.696*	*3.495*	*1.254*	*0.939*	*15.998*	*8.342*	*2.099*	*1.030*	*-25.546*	*-28.011*
2057	*2.130*	*1.839*	*30.282*	*13.189*	*0.574*	*-0.054*	*-34.805*	*-30.812*	*2.615*	*2.862*	*25.129*	*2.872*	*1.246*	*0.928*	*15.492*	*8.038*	*2.094*	*1.014*	*-26.647*	*-28.989*
2058	*2.114*	*1.827*	*29.805*	*12.823*	*0.546*	*-0.089*	*-36.196*	*-31.935*	*2.615*	*2.867*	*24.562*	*2.250*	*1.238*	*0.918*	*14.986*	*7.733*	*2.089*	*0.997*	*-27.743*	*-29.963*
2059	*2.098*	*1.818*	*29.327*	*12.457*	*0.517*	*-0.124*	*-37.584*	*-33.059*	*2.615*	*2.873*	*23.995*	*1.627*	*1.230*	*0.908*	*14.480*	*7.429*	*2.083*	*0.980*	*-28.835*	*-30.936*
2060	*2.082*	*1.807*	*28.850*	*12.091*	*0.488*	*-0.159*	*-38.972*	*-34.183*	*2.615*	*2.879*	*23.429*	*1.004*	*1.222*	*0.897*	*13.973*	*7.124*	*2.078*	*0.963*	*-29.922*	*-31.906*
2061	*2.066*	*1.798*	*28.373*	*11.726*	*0.460*	*-0.194*	*-40.357*	*-35.307*	*2.616*	*2.885*	*22.862*	*0.381*	*1.214*	*0.887*	*13.467*	*6.820*	*2.073*	*0.946*	*-31.005*	*-32.874*
2062	*2.050*	*1.787*	*27.896*	*11.361*	*0.431*	*-0.229*	*-41.742*	*-36.431*	*2.616*	*2.890*	*22.295*	*-0.241*	*1.206*	*0.877*	*12.961*	*6.515*	*2.068*	*0.929*	*-32.084*	*-33.840*
2063	*2.034*	*1.778*	*27.419*	*10.996*	*0.403*	*-0.264*	*-43.125*	*-37.555*	*2.616*	*2.896*	*21.728*	*-0.864*	*1.198*	*0.866*	*12.455*	*6.211*	*2.063*	*0.912*	*-33.160*	*-34.804*
2064	*2.018*	*1.766*	*26.942*	*10.631*	*0.374*	*-0.300*	*-44.507*	*-38.678*	*2.616*	*2.902*	*21.161*	*-1.487*	*1.190*	*0.856*	*11.949*	*5.906*	*2.058*	*0.895*	*-34.231*	*-35.765*
2065	*2.002*	*1.758*	*26.466*	*10.267*	*0.346*	*-0.335*	*-45.888*	*-39.802*	*2.617*	*2.907*	*20.594*	*-2.110*	*1.182*	*0.846*	*11.443*	*5.602*	*2.052*	*0.878*	*-35.298*	*-36.725*
2066	*1.986*	*1.746*	*25.989*	*9.903*	*0.317*	*-0.370*	*-47.267*	*-40.926*	*2.617*	*2.913*	*20.027*	*-2.732*	*1.174*	*0.835*	*10.937*	*5.297*	*2.047*	*0.861*	*-36.361*	*-37.682*
2067	*1.970*	*1.737*	*25.512*	*9.539*	*0.288*	*-0.405*	*-48.646*	*-42.050*	*2.617*	*2.919*	*19.460*	*-3.355*	*1.166*	*0.825*	*10.431*	*4.993*	*2.042*	*0.845*	*-37.421*	*-38.637*
2068	*1.954*	*1.726*	*25.036*	*9.175*	*0.260*	*-0.440*	*-50.024*	*-43.174*	*2.617*	*2.924*	*18.894*	*-3.978*	*1.158*	*0.815*	*9.925*	*4.688*	*2.037*	*0.828*	*-38.477*	*-39.590*
2069	*1.938*	*1.717*	*24.559*	*8.811*	*0.231*	*-0.475*	*-51.401*	*-44.298*	*2.618*	*2.930*	*18.327*	*-4.601*	*1.149*	*0.804*	*9.418*	*4.384*	*2.032*	*0.811*	*-39.530*	*-40.541*
2070	*1.922*	*1.706*	*24.082*	*8.447*	*0.203*	*-0.510*	*-52.777*	*-45.421*	*2.618*	*2.936*	*17.760*	*-5.223*	*1.141*	*0.794*	*8.912*	*4.079*	*2.027*	*0.794*	*-40.578*	*-41.489*
2071	*1.906*	*1.697*	*23.606*	*8.084*	*0.174*	*-0.545*	*-54.153*	*-46.545*	*2.618*	*2.941*	*17.193*	*-5.846*	*1.133*	*0.784*	*8.406*	*3.775*	*2.022*	*0.777*	*-41.624*	*-42.436*
2072	*1.890*	*1.686*	*23.129*	*7.720*	*0.146*	*-0.581*	*-55.528*	*-47.669*	*2.618*	*2.947*	*16.626*	*-6.469*	*1.125*	*0.773*	*7.900*	*3.470*	*2.016*	*0.760*	*-42.666*	*-43.381*
2073	*1.873*	*1.677*	*22.653*	*7.357*	*0.117*	*-0.616*	*-56.902*	*-48.793*	*2.619*	*2.953*	*16.059*	*-7.092*	*1.117*	*0.763*	*7.394*	*3.166*	*2.011*	*0.744*	*-43.705*	*-44.323*
2074	*1.857*	*1.665*	*22.176*	*6.994*	*0.088*	*-0.651*	*-58.275*	*-49.917*	*2.619*	*2.959*	*15.492*	*-7.714*	*1.109*	*0.753*	*6.888*	*2.861*	*2.006*	*0.727*	*-44.740*	*-45.264*
2075	*1.841*	*1.657*	*21.700*	*6.630*	*0.060*	*-0.686*	*-59.648*	*-51.041*	*2.619*	*2.964*	*14.926*	*-8.337*	*1.101*	*0.742*	*6.382*	*2.557*	*2.001*	*0.710*	*-45.772*	*-46.203*
2076	*1.825*	*1.645*	*21.223*	*6.267*	*0.031*	*-0.721*	*-61.021*	*-52.164*	*2.619*	*2.970*	*14.359*	*-8.960*	*1.093*	*0.732*	*5.876*	*2.252*	*1.996*	*0.693*	*-46.802*	*-47.139*
2077	*1.809*	*1.636*	*20.747*	*5.904*	*0.003*	*-0.756*	*-62.393*	*-53.288*	*2.620*	*2.976*	*13.792*	*-9.583*	*1.085*	*0.722*	*5.370*	*1.948*	*1.991*	*0.677*	*-47.828*	*-48.074*
2078	*1.793*	*1.625*	*20.271*	*5.541*	*-0.026*	*-0.791*	*-63.764*	*-54.412*	*2.620*	*2.981*	*13.225*	*-10.205*	*1.077*	*0.712*	*4.863*	*1.643*	*1.986*	*0.660*	*-48.851*	*-49.006*
2079	*1.777*	*1.616*	*19.794*	*5.178*	*-0.055*	*-0.827*	*-65.135*	*-55.536*	*2.620*	*2.987*	*12.658*	*-10.828*	*1.069*	*0.701*	*4.357*	*1.339*	*1.981*	*0.643*	*-49.871*	*-49.937*

2080	*1.761*	*1.605*	*19.318*	*4.816*	*-0.083*	*-0.862*	*-66.506*	*-56.660*	*2.620*	*2.993*	*12.091*	*-11.451*	*1.061*	*0.691*	*3.851*	*1.034*	*1.975*	*0.626*	*-50.888*	*-50.866*
2081	*1.745*	*1.596*	*18.841*	*4.453*	*-0.112*	*-0.897*	*-67.876*	*-57.784*	*2.621*	*2.998*	*11.524*	*-12.073*	*1.053*	*0.681*	*3.345*	*0.730*	*1.970*	*0.610*	*-51.902*	*-51.793*
2082	*1.729*	*1.585*	*18.365*	*4.090*	*-0.140*	*-0.932*	*-69.246*	*-58.907*	*2.621*	*3.004*	*10.957*	*-12.696*	*1.045*	*0.670*	*2.839*	*0.425*	*1.965*	*0.593*	*-52.914*	*-52.718*
2083	*1.713*	*1.576*	*17.889*	*3.728*	*-0.169*	*-0.967*	*-70.615*	*-60.031*	*2.621*	*3.010*	*10.391*	*-13.319*	*1.036*	*0.660*	*2.333*	*0.121*	*1.960*	*0.576*	*-53.923*	*-53.641*
2084	*1.697*	*1.564*	*17.412*	*3.365*	*-0.197*	*-1.002*	*-71.985*	*-61.155*	*2.621*	*3.015*	*9.824*	*-13.942*	*1.028*	*0.650*	*1.827*	*-0.184*	*1.955*	*0.559*	*-54.929*	*-54.562*
2085	*1.681*	*1.555*	*16.936*	*3.002*	*-0.226*	*-1.037*	*-73.354*	*-62.279*	*2.622*	*3.021*	*9.257*	*-14.564*	*1.020*	*0.639*	*1.321*	*-0.488*	*1.950*	*0.543*	*-55.932*	*-55.482*
2086	*1.665*	*1.544*	*16.459*	*2.640*	*-0.255*	*-1.073*	*-74.722*	*-63.403*	*2.622*	*3.027*	*8.690*	*-15.187*	*1.012*	*0.629*	*0.815*	*-0.792*	*1.945*	*0.526*	*-56.933*	*-56.399*
2087	*1.649*	*1.535*	*15.983*	*2.277*	*-0.283*	*-1.108*	*-76.091*	*-64.527*	*2.622*	*3.033*	*8.123*	*-15.810*	*1.004*	*0.619*	*0.308*	*-1.097*	*1.940*	*0.509*	*-57.931*	*-57.315*
2088	*1.633*	*1.524*	*15.507*	*1.915*	*-0.312*	*-1.143*	*-77.459*	*-65.650*	*2.622*	*3.038*	*7.556*	*-16.433*	*0.996*	*0.608*	*-0.198*	*-1.401*	*1.935*	*0.493*	*-58.927*	*-58.229*
2089	*1.616*	*1.515*	*15.030*	*1.553*	*-0.340*	*-1.178*	*-78.827*	*-66.774*	*2.623*	*3.044*	*6.989*	*-17.055*	*0.988*	*0.598*	*-0.704*	*-1.706*	*1.930*	*0.476*	*-59.921*	*-59.141*
2090	*1.600*	*1.504*	*14.554*	*1.190*	*-0.369*	*-1.213*	*-80.195*	*-67.898*	*2.623*	*3.050*	*6.423*	*-17.678*	*0.980*	*0.588*	*-1.210*	*-2.010*	*1.924*	*0.459*	*-60.912*	*-60.051*
2091	*1.584*	*1.495*	*14.078*	*0.828*	*-0.397*	*-1.248*	*-81.562*	*-69.022*	*2.623*	*3.055*	*5.856*	*-18.301*	*0.972*	*0.577*	*-1.716*	*-2.315*	*1.919*	*0.443*	*-61.900*	*-60.960*
2092	*1.568*	*1.483*	*13.601*	*0.465*	*-0.426*	*-1.283*	*-82.929*	*-70.146*	*2.623*	*3.061*	*5.289*	*-18.924*	*0.964*	*0.567*	*-2.222*	*-2.619*	*1.914*	*0.426*	*-62.887*	*-61.866*
2093	*1.552*	*1.475*	*13.125*	*0.103*	*-0.455*	*-1.318*	*-84.297*	*-71.270*	*2.624*	*3.067*	*4.722*	*-19.546*	*0.956*	*0.557*	*-2.728*	*-2.924*	*1.909*	*0.409*	*-63.871*	*-62.771*
2094	*1.536*	*1.463*	*12.649*	*-0.259*	*-0.483*	*-1.354*	*-85.664*	*-72.393*	*2.624*	*3.072*	*4.155*	*-20.169*	*0.948*	*0.546*	*-3.234*	*-3.228*	*1.904*	*0.393*	*-64.853*	*-63.675*
2095	*1.520*	*1.454*	*12.172*	*-0.621*	*-0.512*	*-1.389*	*-87.031*	*-73.517*	*2.624*	*3.078*	*3.588*	*-20.792*	*0.940*	*0.536*	*-3.741*	*-3.533*	*1.899*	*0.376*	*-65.833*	*-64.576*
2096	*1.504*	*1.443*	*11.696*	*-0.984*	*-0.540*	*-1.424*	*-88.397*	*-74.641*	*2.624*	*3.084*	*3.021*	*-21.415*	*0.931*	*0.526*	*-4.247*	*-3.837*	*1.894*	*0.359*	*-66.810*	*-65.476*
2097	*1.488*	*1.434*	*11.220*	*-1.346*	*-0.569*	*-1.459*	*-89.764*	*-75.765*	*2.625*	*3.089*	*2.454*	*-22.037*	*0.923*	*0.515*	*-4.753*	*-4.142*	*1.889*	*0.343*	*-67.786*	*-66.374*
2098	*1.472*	*1.423*	*10.743*	*-1.708*	*-0.598*	*-1.494*	*-91.130*	*-76.889*	*2.625*	*3.095*	*1.888*	*-22.660*	*0.915*	*0.505*	*-5.259*	*-4.446*	*1.884*	*0.326*	*-68.759*	*-67.271*
2099	*1.456*	*1.414*	*10.267*	*-2.070*	*-0.626*	*-1.529*	*-92.497*	*-78.013*	*2.625*	*3.101*	*1.321*	*-23.283*	*0.907*	*0.495*	*-5.765*	*-4.751*	*1.879*	*0.309*	*-69.731*	*-68.166*
2100	*1.440*	*1.403*	*9.791*	*-2.433*	*-0.655*	*-1.564*	*-93.863*	*-79.137*	*2.625*	*3.107*	*0.754*	*-23.906*	*0.899*	*0.484*	*-6.271*	*-5.055*	*1.873*	*0.293*	*-70.700*	*-69.059*
2101	*1.424*	*1.394*	*9.314*	*-2.795*	*-0.683*	*-1.600*	*-95.229*	*-80.260*	*2.626*	*3.112*	*0.187*	*-24.528*	*0.891*	*0.474*	*-6.777*	*-5.360*	*1.868*	*0.276*	*-71.668*	*-69.950*
2102	*1.408*	*1.382*	*8.838*	*-3.157*	*-0.712*	*-1.635*	*-96.595*	*-81.384*	*2.626*	*3.118*	*-0.380*	*-25.151*	*0.883*	*0.464*	*-7.283*	*-5.664*	*1.863*	*0.260*	*-72.634*	*-70.840*
2103	*1.392*	*1.373*	*8.362*	*-3.519*	*-0.740*	*-1.670*	*-97.961*	*-82.508*	*2.626*	*3.124*	*-0.947*	*-25.774*	*0.875*	*0.454*	*-7.789*	*-5.969*	*1.858*	*0.243*	*-73.598*	*-71.729*
2104	*1.376*	*1.362*	*7.885*	*-3.881*	*-0.769*	*-1.705*	*-99.327*	*-83.632*	*2.626*	*3.129*	*-1.514*	*-26.397*	*0.867*	*0.443*	*-8.296*	*-6.273*	*1.853*	*0.226*	*-74.560*	*-72.615*
2105	*1.360*	*1.353*	*7.409*	*-4.243*	*-0.798*	*-1.740*	*-100.693*	*-84.756*	*2.627*	*3.135*	*-2.080*	*-27.019*	*0.859*	*0.433*	*-8.802*	*-6.578*	*1.848*	*0.210*	*-75.520*	*-73.500*
2106	*1.343*	*1.342*	*6.933*	*-4.606*	*-0.826*	*-1.775*	*-102.059*	*-85.880*	*2.627*	*3.141*	*-2.647*	*-27.642*	*0.851*	*0.423*	*-9.308*	*-6.882*	*1.843*	*0.193*	*-76.478*	*-74.384*

2107	*1.327*	*1.333*	*6.456*	*-4.968*	*-0.855*	*-1.810*	*-103.424*	*-87.003*	*2.627*	*3.146*	*-3.214*	*-28.265*	*0.843*	*0.412*	*-9.814*	*-7.187*	*1.838*	*0.176*	*-77.435*	*-75.266*
2108	*1.311*	*1.322*	*5.980*	*-5.330*	*-0.883*	*-1.846*	*-104.790*	*-88.127*	*2.627*	*3.152*	*-3.781*	*-28.888*	*0.835*	*0.402*	*-10.320*	*-7.491*	*1.833*	*0.160*	*-78.390*	*-76.146*
2109	*1.295*	*1.313*	*5.504*	*-5.692*	*-0.912*	*-1.881*	*-106.156*	*-89.251*	*2.628*	*3.158*	*-4.348*	*-29.510*	*0.826*	*0.392*	*-10.826*	*-7.796*	*1.828*	*0.143*	*-79.344*	*-77.025*
2110	*1.279*	*1.302*	*5.027*	*-6.054*	*-0.941*	*-1.916*	*-107.521*	*-90.375*	*2.628*	*3.163*	*-4.915*	*-30.133*	*0.818*	*0.381*	*-11.332*	*-8.100*	*1.822*	*0.127*	*-80.295*	*-77.902*

(Abbreviations: CRC, colorectal cancer; SDI, sociodemographic index)
Note: The numbers in normal font are the data downloaded from GBD and GLOBOCAN. The italicized numbers indicate the forecasted death rates

Supplemental Table 5. The average annual rates of change in DALY rates

Region	Group	Gender	Average AROC of available data	Average AROC of available data and 30-year forecast	Average AROC of available data and 50-year forecast	Average AROC of available data and 90-year forecast
High SDI	YOCRC	Male	-0·16%	-0·38%	-0·46%	-0·60%
		Female	-0·30%	-0·38%	-0·42%	-0·49%
	LOCRC	Male	-1·14%	-1·14%	-1·23%	-1·81%
		Female	-1·71%	-1·68%	-1·89%	4·50%
Australia	YOCRC	Male	-0·61%	-0·53%	-0·52%	-0·55%
		Female	-0·60%	-0·53%	-0·53%	-0·56%
	LOCRC	Male	-2·71%	-3·38%	-3·66%	-0·75%
		Female	-3·30%	-8·09%	-3·47%	-1·45%
Singapore	YOCRC	Male	-2·88%	-5·85%	-2·16%	-0·42%
		Female	-2·69%	-4·17%	-3·71%	-1·26%
	LOCRC	Male	-3·01%	-5·65%	-1·16%	0·09%
		Female	-2·38%	-3·16%	-13·19%	-7·03%
Switzerland	YOCRC	Male	-0·79%	-0·84%	-0·91%	-1·21%
		Female	-0·21%	-0·72%	-0·97%	-2·05%
	LOCRC	Male	-1·09%	-1·66%	-2·27%	0·68%
		Female	-0·88%	-1·01%	-1·14%	-1·83%
USA	YOCRC	Male	0·73%	0·48%	0·42%	0·35%
		Female	0·53%	0·21%	0·13%	0·06%
	LOCRC	Male	-1·60%	-1·09%	-1·00%	-0·99%
		Female	-1·98%	-1·59%	-1·61%	-2·43%

(Abbreviations: AROC, Annual rate of change; DALY, Disability-adjusted life year; LOCRC, Late-onset colorectal cancer; SDI, Socio-demographic index; YOCRC, Young-onset colorectal cancer)

Supplemental Table 6. DALY rate data and forecasts

Year	High SDI				Australia				Singapore				Switzerland				United States			
	Young Onset CRC		Late Onset CRC		Young Onset CRC		Late Onset CRC		Young Onset CRC		Late Onset CRC		Young Onset CRC		Late Onset CRC		Young Onset CRC		Late Onset CRC	
	Male	Female	Male	Female	Male	Female	Male	Female	Male	Female	Male	Female	Male	Female	Male	Female	Male	Female	Male	Female
1990	141.894	119.136	1009.679	685.453	144.527	132.192	1228.041	860.879	146.108	134.549	1143.380	771.848	109.681	74.023	705.764	428.500	131.901	108.436	1048.137	727.292
1991	142.916	119.674	1014.779	683.622	143.123	131.266	1219.137	846.951	148.336	127.048	1149.807	737.644	118.386	75.787	728.492	430.752	133.388	110.047	1034.369	716.927
1992	144.212	119.918	1023.381	681.183	142.617	131.224	1226.607	841.403	151.124	129.102	1111.372	738.467	120.387	78.279	729.945	431.087	136.474	111.132	1022.172	702.705
1993	146.073	121.479	1036.694	685.180	142.190	128.259	1219.886	821.262	153.976	128.643	1100.049	741.612	120.399	82.028	725.866	435.402	140.875	114.423	1025.320	701.082
1994	146.760	121.455	1036.377	677.783	144.100	129.698	1230.518	820.804	155.540	126.641	1095.832	741.081	123.968	85.012	732.620	431.501	143.440	116.893	1013.498	689.451
1995	148.251	123.012	1038.616	672.066	143.845	127.746	1202.947	797.005	153.394	129.999	1085.404	734.294	114.591	85.120	778.454	454.119	146.055	118.786	1000.359	679.091
1996	148.390	123.240	1024.666	657.225	142.543	129.402	1193.310	781.982	149.166	131.550	1082.665	735.298	109.839	82.948	762.367	446.872	146.253	119.627	966.268	659.980
1997	148.136	122.858	998.474	639.174	141.562	130.924	1148.987	757.084	134.035	130.279	1035.206	718.843	109.352	83.718	760.793	452.910	146.140	120.609	922.185	636.796
1998	147.605	121.988	986.069	626.335	141.083	133.123	1105.648	721.756	126.543	129.425	1013.257	693.419	106.504	83.750	766.018	439.990	144.775	120.365	896.465	618.236
1999	147.290	121.958	973.230	617.523	135.437	133.564	1057.224	682.585	115.104	122.673	960.310	662.282	101.450	82.834	754.060	442.646	146.849	123.027	880.973	610.711
2000	149.159	122.065	952.492	605.270	134.709	131.521	1009.136	663.900	113.188	117.107	909.270	648.263	106.843	84.321	757.834	461.449	151.160	125.291	858.720	594.420
2001	150.578	122.477	933.704	592.107	128.997	127.474	953.634	639.275	113.702	110.031	903.344	640.646	107.123	85.644	740.674	442.684	155.060	129.580	838.943	581.267
2002	151.240	121.572	920.919	579.535	125.651	125.928	899.897	620.912	113.558	104.751	879.554	633.276	107.326	88.026	724.994	433.233	157.656	130.919	824.757	563.292
2003	151.696	121.169	908.084	567.581	120.804	125.257	830.609	583.532	110.100	103.231	844.161	602.024	104.633	86.495	716.781	422.118	157.054	130.862	797.592	545.076
2004	150.675	119.859	884.366	548.588	122.590	121.479	832.851	557.763	102.487	106.658	797.467	596.920	107.228	85.716	698.795	402.464	153.318	129.074	759.164	519.362
2005	150.724	119.408	871.663	536.104	123.041	116.320	816.958	532.632	98.055	103.226	772.775	543.475	107.182	86.938	684.724	388.945	154.371	130.006	744.257	506.247
2006	149.930	118.102	852.851	521.930	121.248	115.131	795.352	516.375	95.856	101.452	728.244	534.385	102.976	85.167	669.975	389.562	155.109	130.097	724.943	493.016
2007	148.956	117.223	843.623	512.506	122.050	116.586	794.139	516.070	92.226	93.499	715.664	515.516	101.401	80.935	664.852	391.685	154.959	128.211	713.183	479.912
2008	149.001	117.786	838.135	504.831	124.731	115.546	782.538	502.858	91.396	89.707	699.019	515.578	102.403	77.869	641.468	383.626	157.221	129.176	713.005	473.397
2009	148.591	117.901	830.929	495.969	127.738	117.724	760.028	489.540	86.846	83.958	685.678	491.294	103.030	76.889	636.387	378.983	159.158	129.998	710.349	468.998
2010	146.470	116.190	821.104	487.071	127.527	115.387	743.152	472.954	83.466	81.762	668.340	486.962	100.433	79.542	621.985	374.902	158.509	126.952	701.607	458.797
2011	144.480	115.482	813.031	482.979	123.736	115.608	716.645	459.752	82.861	82.440	652.948	474.901	97.756	79.068	604.933	362.681	158.947	126.546	702.397	457.470
2012	141.75	113.71	801.853	475.12	122.07	112.68	688.427	443.03	79.052	83.245	636.258	482.84	94.011	77.590	590.20	363.28	158.00	125.28	702.237	453.99

	1	8		8	9	6		1				3			4	4	7	1		2
2013	140.104	112.660	793.006	470.040	124.261	114.325	680.784	433.990	79.028	76.103	622.422	464.841	93.725	73.375	580.800	356.635	157.733	124.563	704.190	453.821
2014	139.471	111.828	766.059	448.013	123.261	113.060	627.712	392.386	71.812	70.037	560.885	430.002	89.437	71.728	556.443	347.368	159.839	126.457	678.904	432.604
2015	138.407	111.295	757.046	441.401	122.908	112.962	611.842	377.891	69.792	67.339	541.154	416.686	88.730	70.945	545.943	343.413	160.271	126.201	675.558	428.135
2016	137.418	110.629	748.295	434.836	122.424	112.159	595.746	362.914	67.646	65.577	521.598	409.509	88.055	70.404	535.619	339.652	160.706	125.962	672.190	423.660
2017	136.490	110.199	739.775	428.415	121.957	111.976	580.299	348.755	65.629	63.336	502.219	397.111	87.379	69.832	525.522	335.984	161.135	125.737	668.696	419.213
2018	135.608	109.594	731.462	422.081	121.433	111.228	565.036	334.367	63.563	61.839	483.007	390.248	86.702	69.172	515.619	332.408	161.555	125.526	665.164	414.783
2019	134.762	109.200	723.332	415.848	120.940	110.984	550.118	320.528	61.555	59.750	463.957	378.390	86.025	68.461	505.901	328.906	161.971	125.326	661.563	410.371
2020	*133.944*	*108.617*	*715.364*	*409.701*	*120.412*	*110.285*	*535.423*	*306.604*	*59.528*	*58.341*	*445.062*	*371.596*	*85.348*	*67.743*	*496.352*	*325.465*	*162.385*	*125.137*	*657.930*	*405.977*
2021	*133.147*	*108.236*	*707.540*	*403.636*	*119.910*	*109.995*	*520.963*	*293.058*	*57.528*	*56.303*	*426.315*	*360.086*	*84.671*	*67.035*	*486.962*	*322.075*	*162.799*	*124.959*	*654.257*	*401.599*
2022	*132.367*	*107.659*	*699.843*	*397.647*	*119.385*	*109.337*	*506.696*	*279.504*	*55.520*	*54.923*	*407.712*	*353.248*	*83.994*	*66.335*	*477.718*	*318.728*	*163.214*	*124.789*	*650.560*	*397.236*
2023	*131.599*	*107.283*	*692.260*	*391.729*	*118.879*	*109.010*	*492.607*	*266.217*	*53.525*	*52.903*	*389.245*	*341.985*	*83.317*	*65.638*	*468.610*	*315.416*	*163.629*	*124.627*	*646.839*	*392.887*
2024	*130.841*	*106.709*	*684.778*	*385.876*	*118.356*	*108.384*	*478.674*	*252.963*	*51.527*	*51.533*	*370.911*	*335.053*	*82.639*	*64.940*	*459.629*	*312.134*	*164.044*	*124.472*	*643.101*	*388.552*
2025	*130.091*	*106.334*	*677.385*	*380.083*	*117.848*	*108.027*	*464.880*	*239.903*	*49.535*	*49.518*	*352.703*	*323.981*	*81.962*	*64.241*	*450.764*	*308.875*	*164.459*	*124.324*	*639.349*	*384.230*
2026	*129.347*	*105.762*	*670.071*	*374.347*	*117.327*	*107.429*	*451.209*	*226.892*	*47.542*	*48.151*	*334.617*	*316.935*	*81.285*	*63.541*	*442.009*	*305.637*	*164.875*	*124.182*	*635.585*	*379.919*
2027	*128.607*	*105.387*	*662.828*	*368.664*	*116.816*	*107.048*	*437.648*	*214.027*	*45.553*	*46.138*	*316.648*	*306.024*	*80.608*	*62.842*	*433.354*	*302.416*	*165.290*	*124.045*	*631.812*	*375.620*
2028	*127.870*	*104.815*	*655.647*	*363.028*	*116.297*	*106.471*	*424.184*	*201.218*	*43.563*	*44.772*	*298.791*	*298.857*	*79.931*	*62.142*	*424.793*	*299.209*	*165.705*	*123.913*	*628.032*	*371.331*
2029	*127.136*	*104.440*	*648.522*	*357.438*	*115.785*	*106.070*	*410.806*	*188.519*	*41.574*	*42.760*	*281.041*	*288.086*	*79.254*	*61.443*	*416.319*	*296.014*	*166.121*	*123.786*	*624.245*	*367.053*
2030	*126.404*	*103.868*	*641.445*	*351.890*	*115.267*	*105.512*	*397.505*	*175.876*	*39.585*	*41.395*	*263.395*	*280.802*	*78.577*	*60.744*	*407.926*	*292.828*	*166.536*	*123.663*	*620.454*	*362.784*
2031	*125.674*	*103.494*	*634.413*	*346.380*	*114.754*	*105.095*	*384.273*	*163.319*	*37.597*	*39.382*	*245.849*	*270.157*	*77.900*	*60.044*	*399.608*	*289.650*	*166.951*	*123.543*	*616.659*	*358.523*
2032	*124.945*	*102.922*	*627.419*	*340.907*	*114.237*	*104.550*	*371.101*	*150.813*	*35.609*	*38.017*	*228.397*	*262.759*	*77.223*	*59.345*	*391.359*	*286.479*	*167.366*	*123.426*	*612.860*	*354.271*
2033	*124.217*	*102.548*	*620.459*	*335.467*	*113.724*	*104.121*	*357.982*	*138.376*	*33.622*	*36.005*	*211.038*	*252.230*	*76.546*	*58.645*	*383.175*	*283.313*	*167.782*	*123.312*	*609.060*	*350.027*
2034	*123.490*	*101.975*	*613.531*	*330.058*	*113.207*	*103.588*	*344.912*	*125.985*	*31.634*	*34.640*	*193.766*	*244.724*	*75.869*	*57.946*	*375.051*	*280.152*	*168.197*	*123.201*	*605.257*	*345.790*
2035	*122.763*	*101.601*	*606.629*	*324.677*	*112.693*	*103.148*	*331.884*	*113.649*	*29.647*	*32.628*	*176.579*	*234.302*	*75.192*	*57.247*	*366.982*	*276.995*	*168.612*	*123.093*	*601.452*	*341.560*
2036	*122.037*	*101.029*	*599.751*	*319.324*	*112.177*	*102.624*	*318.894*	*101.354*	*27.660*	*31.263*	*159.473*	*226.696*	*74.515*	*56.547*	*358.965*	*273.841*	*169.027*	*122.986*	*597.646*	*337.337*
2037	*121.311*	*100.655*	*592.894*	*313.995*	*111.662*	*102.175*	*305.937*	*89.103*	*25.672*	*29.251*	*142.446*	*216.371*	*73.838*	*55.848*	*350.996*	*270.690*	*169.443*	*122.882*	*593.839*	*333.120*
2038	*120.585*	*100.083*	*586.057*	*308.690*	*111.146*	*101.659*	*293.010*	*76.887*	*23.685*	*27.885*	*125.493*	*208.671*	*73.161*	*55.149*	*343.071*	*267.540*	*169.858*	*122.779*	*590.031*	*328.909*

2039	119.860	99.708	579.236	303.406	110.632	101.204	280.109	64.709	21.698	25.873	108.612	198.439	72.484	54.449	335.187	264.393	170.273	122.678	586.223	324.703
2040	119.134	99.136	572.430	298.141	110.116	100.694	267.232	52.560	19.711	24.508	91.800	190.651	71.807	53.750	327.342	261.247	170.688	122.579	582.414	320.502
2041	118.409	98.762	565.637	292.895	109.601	100.233	254.375	40.441	17.724	22.496	75.054	180.503	71.129	53.051	319.532	258.103	171.104	122.481	578.604	316.307
2042	117.684	98.190	558.856	287.666	109.086	99.728	241.538	28.348	15.737	21.131	58.372	172.633	70.452	52.351	311.755	254.959	171.519	122.384	574.795	312.116
2043	116.959	97.816	552.086	282.453	108.571	99.263	228.716	16.281	13.750	19.119	41.752	162.565	69.775	51.652	304.009	251.817	171.934	122.288	570.984	307.929
2044	116.234	97.244	545.324	277.255	108.055	98.762	215.909	4.234	11.762	17.754	25.190	154.619	69.098	50.953	296.291	248.675	172.349	122.193	567.174	303.746
2045	115.509	96.869	538.571	272.071	107.540	98.293	203.116	-7.791	9.775	15.742	8.684	144.624	68.421	50.253	288.600	245.533	172.765	122.099	563.363	299.567
2046	114.784	96.297	531.826	266.899	107.025	97.796	190.333	-19.798	7.788	14.376	-7.767	136.607	67.744	49.554	280.932	242.393	173.180	122.005	559.553	295.392
2047	114.059	95.923	525.086	261.739	106.510	97.324	177.561	-31.787	5.801	12.364	-24.166	126.681	67.067	48.854	273.288	239.252	173.595	121.913	555.742	291.220
2048	113.334	95.351	518.353	256.590	105.994	96.829	164.798	-43.761	3.814	10.999	-40.516	118.598	66.390	48.155	265.665	236.112	174.011	121.821	551.931	287.051
2049	112.609	94.977	511.624	251.451	105.479	96.355	152.043	-55.721	1.827	8.987	-56.817	108.736	65.713	47.456	258.061	232.972	174.426	121.730	548.120	282.885
2050	111.885	94.405	504.900	246.321	104.964	95.862	139.296	-67.668	-0.160	7.622	-73.072	100.590	65.036	46.756	250.476	229.833	174.841	121.639	544.309	278.722
2051	111.160	94.030	498.180	241.200	104.449	95.386	126.554	-79.602	-2.147	5.610	-89.283	90.788	64.359	46.057	242.907	226.693	175.256	121.549	540.498	274.562
2052	110.435	93.458	491.464	236.088	103.934	94.894	113.819	-91.526	-4.134	4.245	-105.451	82.585	63.682	45.358	235.354	223.554	175.672	121.459	536.687	270.404
2053	109.710	93.084	484.751	230.982	103.418	94.417	101.088	-103.439	-6.121	2.233	-121.578	72.839	63.005	44.658	227.815	220.415	176.087	121.369	532.875	266.249
2054	108.985	92.512	478.040	225.884	102.903	93.927	88.362	-115.343	-8.108	0.867	-137.666	64.582	62.328	43.959	220.290	217.276	176.502	121.280	529.064	262.095
2055	108.260	92.138	471.332	220.793	102.388	93.448	75.639	-127.239	-10.095	-1.145	-153.717	54.887	61.651	43.260	212.777	214.138	176.917	121.192	525.253	257.944
2056	107.536	91.566	464.627	215.707	101.873	92.959	62.921	-139.127	-12.083	-2.510	-169.731	46.581	60.974	42.560	205.276	210.999	177.333	121.103	521.442	253.795
2057	106.811	91.191	457.923	210.627	101.357	92.480	50.205	-151.008	-14.070	-4.522	-185.711	36.934	60.297	41.861	197.786	207.860	177.748	121.015	517.630	249.648
2058	106.086	90.619	451.221	205.552	100.842	91.992	37.492	-162.882	-16.057	-5.887	-201.657	28.581	59.619	41.162	190.306	204.722	178.163	120.927	513.819	245.503
2059	105.361	90.245	444.520	200.482	100.327	91.511	24.782	-174.750	-18.044	-7.899	-217.571	18.979	58.942	40.462	182.835	201.583	178.578	120.839	510.008	241.359
2060	104.636	89.673	437.821	195.416	99.812	91.024	12.074	-186.613	-20.031	-9.264	-233.454	10.583	58.265	39.763	175.373	198.444	178.994	120.752	506.196	237.217
2061	103.912	89.298	431.123	190.355	99.297	90.543	-0.632	-198.47	-22.018	-11.276	-249.308	1.023	57.588	39.063	167.918	195.306	179.409	120.664	502.385	233.076

1

2062	103.187	88.726	424.426	185.298	98.781	90.056	-13.337	-210.325	-24.005	-12.642	-265.133	-7.414	56.911	38.364	160.471	192.167	179.824	120.577	498.574	228.936
2063	102.462	88.352	417.730	180.244	98.266	89.574	-26.040	-222.174	-25.992	-14.653	-280.931	-16.935	56.234	37.665	153.031	189.029	180.239	120.490	494.763	224.798
2064	101.737	87.780	411.035	175.193	97.751	89.088	-38.741	-234.019	-27.979	-16.019	-296.703	-25.409	55.557	36.965	145.597	185.891	180.655	120.403	490.951	220.661
2065	101.012	87.406	404.341	170.145	97.236	88.606	-51.442	-245.861	-29.966	-18.031	-312.450	-34.894	54.880	36.266	138.170	182.752	181.070	120.316	487.140	216.526
2066	100.287	86.834	397.648	165.101	96.720	88.120	-64.141	-257.700	-31.953	-19.396	-328.173	-43.403	54.203	35.567	130.747	179.614	181.485	120.230	483.329	212.391
2067	99.563	86.459	390.955	160.059	96.205	87.638	-76.839	-269.535	-33.940	-21.408	-343.872	-52.854	53.526	34.867	123.330	176.475	181.900	120.143	479.517	208.257
2068	98.838	85.887	384.262	155.019	95.690	87.152	-89.537	-281.369	-35.927	-22.773	-359.549	-61.396	52.849	34.168	115.918	173.337	182.316	120.057	475.706	204.125
2069	98.113	85.513	377.570	149.982	95.175	86.669	-102.233	-293.199	-37.914	-24.785	-375.205	-70.815	52.172	33.469	108.510	170.198	182.731	119.970	471.895	199.993
2070	97.388	84.941	370.879	144.946	94.660	86.184	-114.930	-305.027	-39.902	-26.151	-390.840	-79.388	51.495	32.769	101.106	167.060	183.146	119.884	468.083	195.862
2071	96.663	84.567	364.187	139.913	94.144	85.701	-127.625	-316.854	-41.889	-28.162	-406.455	-88.778	50.818	32.070	93.705	163.922	183.562	119.798	464.272	191.732
2072	95.939	83.995	357.496	134.882	93.629	85.216	-140.320	-328.678	-43.876	-29.528	-422.051	-97.379	50.141	31.371	86.309	160.783	183.977	119.711	460.461	187.602
2073	95.214	83.620	350.806	129.852	93.114	84.733	-153.014	-340.501	-45.863	-31.540	-437.630	-106.741	49.464	30.671	78.915	157.645	184.392	119.625	456.649	183.474
2074	94.489	83.048	344.115	124.824	92.599	84.248	-165.708	-352.322	-47.850	-32.905	-453.190	-115.369	48.787	29.972	71.525	154.507	184.807	119.539	452.838	179.346
2075	93.764	82.674	337.425	119.797	92.083	83.765	-178.402	-364.141	-49.837	-34.917	-468.734	-124.705	48.109	29.272	64.137	151.368	185.223	119.453	449.027	175.218
2076	93.039	82.102	330.735	114.772	91.568	83.280	-191.095	-375.959	-51.824	-36.282	-484.262	-133.358	47.432	28.573	56.752	148.230	185.638	119.367	445.215	171.092
2077	92.314	81.728	324.046	109.748	91.053	82.797	-203.788	-387.776	-53.811	-38.294	-499.774	-142.671	46.755	27.874	49.370	145.092	186.053	119.281	441.404	166.966
2078	91.590	81.156	317.356	104.725	90.538	82.312	-216.481	-399.592	-55.798	-39.660	-515.272	-151.346	46.078	27.174	41.990	141.953	186.468	119.195	437.592	162.840

2079	90.865	80.781	310.667	99.703	90.023	81.829	-229.174	-411.407	-57.785	-41.671	-530.755	-160.636	45.401	26.475	34.611	138.815	186.884	119.109	433.781	158.715
2080	90.140	80.209	303.977	94.683	89.507	81.344	-241.866	-423.221	-59.772	-43.037	-546.224	-169.333	44.724	25.776	27.235	135.676	187.299	119.023	429.970	154.590
2081	89.415	79.835	297.288	89.663	88.992	80.860	-254.558	-435.034	-61.759	-45.049	-561.680	-178.603	44.047	25.076	19.861	132.538	187.714	118.937	426.158	150.466
2082	88.690	79.263	290.599	84.644	88.477	80.376	-267.250	-446.846	-63.746	-46.414	-577.124	-187.320	43.370	24.377	12.488	129.400	188.129	118.852	422.347	146.342
2083	87.966	78.889	283.910	79.626	87.962	79.892	-279.942	-458.658	-65.734	-48.426	-592.555	-196.571	42.693	23.678	5.116	126.261	188.545	118.766	418.536	142.219
2084	87.241	78.317	277.221	74.608	87.446	79.408	-292.634	-470.469	-67.721	-49.791	-607.975	-205.306	42.016	22.978	-2.253	123.123	188.960	118.680	414.724	138.095
2085	86.516	77.942	270.532	69.592	86.931	78.924	-305.325	-482.279	-69.708	-51.803	-623.384	-214.539	41.339	22.279	-9.622	119.985	189.375	118.594	410.913	133.973
2086	85.791	77.370	263.843	64.576	86.416	78.440	-318.017	-494.089	-71.695	-53.169	-638.781	-223.291	40.662	21.580	-16.989	116.846	189.790	118.508	407.102	129.850
2087	85.066	76.996	257.155	59.560	85.901	77.956	-330.708	-505.898	-73.682	-55.180	-654.169	-232.507	39.985	20.880	-24.355	113.708	190.206	118.423	403.290	125.728
2088	84.342	76.424	250.466	54.545	85.386	77.472	-343.400	-517.707	-75.669	-56.546	-669.546	-241.276	39.308	20.181	-31.720	110.570	190.621	118.337	399.479	121.606
2089	83.617	76.049	243.777	49.531	84.870	76.988	-356.091	-529.515	-77.656	-58.558	-684.914	-250.477	38.631	19.481	-39.084	107.431	191.036	118.251	395.668	117.485
2090	82.892	75.478	237.089	44.517	84.355	76.504	-368.782	-541.323	-79.643	-59.923	-700.273	-259.260	37.954	18.782	-46.448	104.293	191.452	118.165	391.856	113.364
2091	82.167	75.103	230.400	39.503	83.840	76.020	-381.473	-553.131	-81.630	-61.935	-715.623	-268.446	37.277	18.083	-53.810	101.155	191.867	118.080	388.045	109.243
2092	81.442	74.531	223.712	34.490	83.325	75.536	-394.164	-564.938	-83.617	-63.300	-730.964	-277.244	36.599	17.383	-61.171	98.016	192.282	117.994	384.234	105.122
2093	80.717	74.157	217.023	29.478	82.810	75.052	-406.855	-576.745	-85.604	-65.312	-746.298	-286.416	35.922	16.684	-68.532	94.878	192.697	117.908	380.422	101.001
2094	79.993	73.585	210.335	24.465	82.294	74.568	-419.547	-588.552	-87.591	-66.678	-761.623	-295.227	35.245	15.985	-75.892	91.739	193.113	117.822	376.611	96.881
2095	79.268	73.210	203.646	19.453	81.779	74.084	-432.238	-600.358	-89.578	-68.689	-776.941	-304.387	34.568	15.285	-83.252	88.601	193.528	117.737	372.800	92.761
2096	78.543	72.638	196.958	14.442	81.264	73.599	-444.928	-612.165	-91.566	-70.055	-792.252	-313.210	33.891	14.586	-90.611	85.463	193.943	117.651	368.988	88.641

2097	*77.818*	*72.264*	*190.269*	*9.430*	*80.749*	*73.115*	*-457.619*	*-623.971*	*-93.553*	*-72.066*	*-807.556*	*-322.358*	*33.214*	*13.887*	*-97.969*	*82.324*	*194.358*	*117.565*	*365.177*	*84.521*
2098	*77.093*	*71.692*	*183.581*	*4.419*	*80.233*	*72.631*	*-470.310*	*-635.777*	*-95.540*	*-73.432*	*-822.854*	*-331.192*	*32.537*	*13.187*	*-105.327*	*79.186*	*194.774*	*117.480*	*361.366*	*80.401*
2099	*76.369*	*71.318*	*176.892*	*-0.592*	*79.718*	*72.147*	*-483.001*	*-647.582*	*-97.527*	*-75.444*	*-838.145*	*-340.330*	*31.860*	*12.488*	*-112.684*	*76.048*	*195.189*	*117.394*	*357.554*	*76.282*
2100	*75.644*	*70.746*	*170.204*	*-5.602*	*79.203*	*71.663*	*-495.692*	*-659.388*	*-99.514*	*-76.809*	*-853.430*	*-349.174*	*31.183*	*11.789*	*-120.041*	*72.909*	*195.604*	*117.308*	*353.743*	*72.162*
2101	*74.919*	*70.371*	*163.515*	*-10.613*	*78.688*	*71.179*	*-508.383*	*-671.193*	*-101.501*	*-78.821*	*-868.709*	*-358.302*	*30.506*	*11.089*	*-127.397*	*69.771*	*196.019*	*117.223*	*349.932*	*68.043*
2102	*74.194*	*69.799*	*156.827*	*-15.623*	*78.173*	*70.695*	*-521.074*	*-682.998*	*-103.488*	*-80.187*	*-883.982*	*-367.156*	*29.829*	*10.390*	*-134.754*	*66.633*	*196.435*	*117.137*	*346.120*	*63.924*
2103	*73.469*	*69.425*	*150.139*	*-20.633*	*77.657*	*70.211*	*-533.765*	*-694.803*	*-105.475*	*-82.198*	*-899.250*	*-376.274*	*29.152*	*9.690*	*-142.109*	*63.494*	*196.850*	*117.051*	*342.309*	*59.805*
2104	*72.745*	*68.853*	*143.450*	*-25.643*	*77.142*	*69.727*	*-546.455*	*-706.608*	*-107.462*	*-83.564*	*-914.513*	*-385.137*	*28.475*	*8.991*	*-149.465*	*60.356*	*197.265*	*116.966*	*338.498*	*55.686*
2105	*72.020*	*68.479*	*136.762*	*-30.653*	*76.627*	*69.243*	*-559.146*	*-718.413*	*-109.449*	*-85.575*	*-929.770*	*-394.246*	*27.798*	*8.292*	*-156.820*	*57.218*	*197.680*	*116.880*	*334.686*	*51.567*
2106	*71.295*	*67.907*	*130.074*	*-35.662*	*76.112*	*68.759*	*-571.837*	*-730.218*	*-111.436*	*-86.941*	*-945.023*	*-403.118*	*27.121*	*7.592*	*-164.175*	*54.079*	*198.096*	*116.794*	*330.875*	*47.448*
2107	*70.570*	*67.532*	*123.385*	*-40.672*	*75.596*	*68.275*	*-584.528*	*-742.023*	*-113.423*	*-88.953*	*-960.272*	*-412.219*	*26.444*	*6.893*	*-171.530*	*50.941*	*198.511*	*116.709*	*327.064*	*43.330*
2108	*69.845*	*66.960*	*116.697*	*-45.681*	*75.081*	*67.791*	*-597.219*	*-753.827*	*-115.410*	*-90.318*	*-975.516*	*-421.099*	*25.767*	*6.194*	*-178.884*	*47.802*	*198.926*	*116.623*	*323.252*	*39.211*
2109	*69.120*	*66.586*	*110.009*	*-50.691*	*74.566*	*67.307*	*-609.909*	*-765.632*	*-117.397*	*-92.330*	*-990.755*	*-430.192*	*25.089*	*5.494*	*-186.238*	*44.664*	*199.342*	*116.537*	*319.441*	*35.093*
2110	*68.396*	*66.014*	*103.320*	*-55.700*	*74.051*	*66.823*	*-622.600*	*-777.436*	*-119.385*	*-93.695*	*-1005.991*	*-439.080*	*24.412*	*4.795*	*-193.592*	*41.526*	*199.757*	*116.452*	*315.630*	*30.975*

(Abbreviations: CRC, colorectal cancer; DALY, disability-adjusted life year; SDI, sociodemographic index)
Note: The numbers in normal font are the data downloaded from GBD and GLOBOCAN. The italicized numbers indicate the forecasted incidence rates

Supplemental Table 7. Performance metrics for each YOCRC forecast

YOCRC Incidence rate forecast	MAE	MSE	RMSE	NRMSE
Males (High SDI regions)	0·0395	0·0087	0·0934	0·0282
Females (High SDI regions)	0·0234	0·0035	0·0592	0·0218
Males (Australia)	0·1877	0·2127	0·4612	0·0942
Females (Australia)	0·1858	0·2793	0·5285	0·1079
Males (Singapore)	0·0759	0·0521	0·2284	0·0876
Females (Singapore)	0·0312	0·0080	0·0896	0·0539
Males (Switzerland)	0·2049	0·2270	0·4765	0·2214
Females (Switzerland)	0·0670	0·0306	0·1750	0·0610
Males (USA)	0·1232	0·1086	0·3296	0·0395
Females (USA)	0·0886	0·0861	0·2934	0·0466
**YOCRC Death rate forecast**	**MAE**	**MSE**	**RMSE**	**NRMSE**
Males (High SDI regions)	0·0077	0·0004	0·0201	0·0330
Females (High SDI regions)	0·0089	0·0005	0·0215	0·0469
Males (Australia)	0·0477	0·0203	0·1425	0·0506
Females (Australia)	0·0815	0·0443	0·2104	0·0539
Males (Singapore)	0·1100	0·0914	0·3024	0·1405
Females (Singapore)	0·1254	0·1084	0·3293	0·1293
Males (Switzerland)	0·0367	0·0100	0·1000	0·0471
Females (Switzerland)	0·0483	0·0179	0·1339	0·0665
Males (USA)	0·0560	0·0171	0·1307	0·0940
Females (USA)	0·0708	0·0265	0·1628	0·0609
**YOCRC DALY rate forecast**	**MAE**	**MSE**	**RMSE**	**NRMSE**
Males (High SDI regions)	0·3908	1·0477	1·0236	0·0372
Females (High SDI regions)	0·4389	1·1353	1·0655	0·0515
Males (Australia)	1·5453	12·3320	3·5117	0·1140
Females (Australia)	1·1680	7·1536	2·6746	0·0908
Males (Singapore)	2·6252	41·5987	6·4497	0·0529
Females (Singapore)	1·6779	17·1046	4·1358	0·0420
Males (Switzerland)	1·2901	9·7285	3·1191	0·0658
Females (Switzerland)	0·2663	0·7234	0·8505	0·0289
Males (USA)	0·8799	6·6516	2·5791	0·0719
Females (USA)	0·3578	0·9612	0·9804	0·0436

(Abbreviations: YOCRC, Young-onset colorectal cancer; DALY, Disability-adjusted life year; MAE, Mean absolute error; MSE, Mean squared error; RMSE, Root mean squared error; NRMSE, Normalized root mean squared error; SDI, Socio-demographic index)

Supplemental Table 8. Performance metrics for each LOCRC forecast

LOCRC Incidence rate forecast	MAE	MSE	RMSE	NRMSE
Males (High SDI regions)	0·3987	1·3129	1·1458	0·0524
Females (High SDI regions)	0·6862	3·1022	1·7613	0·0628
Males (Australia)	2·4525	34·8787	5·9058	0·1147
Females (Australia)	1·2819	9·7521	3·1228	0·0941
Males (Singapore)	0·4646	2·8088	1·6759	0·0305
Females (Singapore)	0·4752	1·3985	1·1826	0·0498
Males (Switzerland)	0·5914	1·9587	1·3995	0·0287
Females (Switzerland)	0·2474	0·6707	0·8190	0·0316
Males (USA)	0·5287	2·3091	1·5196	0·0137
Females (USA)	0·3529	0·9618	0·9807	0·0130
**LOCRC Death rate forecast**	**MAE**	**MSE**	**RMSE**	**NRMSE**
Males (High SDI regions)	0·5446	1·7938	1·3393	0·0436
Females (High SDI regions)	0·5196	1·5682	1·2523	0·0484
Males (Australia)	0·7559	5·2411	2·2893	0·0304
Females (Australia)	0·8405	4·6211	2·1497	0·0366
Males (Singapore)	0·5462	1·9482	1·3958	0·0240
Females (Singapore)	0·2723	0·6254	0·7908	0·0172
Males (Switzerland)	0·5241	1·9564	1·3987	0·0295
Females (Switzerland)	0·3109	0·7832	0·8850	0·0289
Males (USA)	1·2753	10·5252	3·2443	0·0572
Females (USA)	1·2633	9·5371	3·0882	0·0571
**LOCRC DALY rate forecast**	**MAE**	**MSE**	**RMSE**	**NRMSE**
Males (High SDI regions)	7·1085	308·7935	17·5725	0·0420
Females (High SDI regions)	6·9044	277·3731	16·6545	0·0476
Males (Australia)	22·1502	2810·0933	53·0103	0·0608
Females (Australia)	17·3668	1764·9775	42·0116	0·0581
Males (Singapore)	15·5600	1450·2437	38·0821	0·0406
Females (Singapore)	5·2053	177·0128	13·3046	0·0256
Males (Switzerland)	2·2388	30·6862	5·5395	0·0140
Females (Switzerland)	1·6807	19·5941	4·4265	0·0248
Males (USA)	11·9866	852·6030	29·1994	0·0665
Females (USA)	9·4594	500·6424	22·3750	0·0593

(Abbreviations: LOCRC, Late-onset colorectal cancer; DALY, Disability-adjusted life year; MAE, Mean absolute error; MSE, Mean squared error; RMSE, Root mean squared error; NRMSE, Normalized root mean squared error; SDI, Socio-demographic index)

Supplemental References: Studies referred to in the Supplemental material

1. Dahia SS, Konduru L, S.G B. A Systematic Review of Cancer Burden Forecasting Models: Evaluating Efficacy for Long-Term Predictions Using Annual Data, 31 March 2024, PREPRINT (Version 1). *Research Square* 2024.

2. Abeel T, Helleputte T, Van de Peer Y, Dupont P, Saeys Y. Robust biomarker identification for cancer diagnosis with ensemble feature selection methods. *Bioinformatics* 2010; **26**(3):392-8.

3. Sanders RE. The Pareto Principle: Its Use and Abuse. *J Serv Mark* 1987; 1:37-40.
